# Cellular distribution of estrogen receptors alpha in the rabbit ovary during pregnancy and lactation

**DOI:** 10.1038/s41598-025-99582-9

**Published:** 2025-07-02

**Authors:** Mahmoud Abd-Elkareem, Sulaiman Mohammed Alnasser, Alotaibi Meshal, Mohsen A. Khormi, Mohammed A. Alfattah

**Affiliations:** 1 Department of Cell and Tissues, Faculty of Veterinary Medicine, Assiut University, Assiut, 71526 Egypt; 2 Department of Pharmacology and Toxicology, College of Pharmacy, Qassim University, 52571 Buraydah, Kingdom of Saudi Arabia; 3 Pharmacy Practice, College of Pharmacy, University of Hafr Albatin, Hafr Albatin, Kingdom of Saudi Arabia; 4 Department of Biology, College of Science, Jazan University, P.O. Box. 114, 45142 Jazan, Kingdom of Saudi Arabia

**Keywords:** Rabbit, Ovary, ERA, Follicles, CL, Immunolocalization, Cell biology, Physiology, Structural biology, Anatomy, Endocrinology

## Abstract

Pregnancy and lactation is a critical period for rabbit production. Estrogen (E2) and estrogen receptors alpha (ERA) are essential during pregnancy and lactation and their importance stems from their role in ovarian activities. Despite extensive research into the roles of E2 and its receptors in the ovary, cellular distribution of ERA in the rabbit ovary during pregnancy, after parturition and during lactation remained unexpectedly elusive. To achieve this aim, eighteen healthy sexually mature New Zealand white rabbit does (2.97 ± 0.2 kg) were raised in the animal house, faculty of medicine, Assiut University. The females rabbit were mated by fertile bucks; the day of mating as was considered Day 0 of pregnancy. Ovaries were collected at 12 h, 3, 7, 14 days post-mating, at parturition and at 10 days of lactation and fixed then processed for immunohistochemistry of ERA. In the present study, the cellular distribution of ERA in the rabbit ovary during pregnancy, postpartum and during lactation revealed moderate ERA immunolocalization in the ovarian surface epithelial cells, stroma cells, fibroblast cells of the tunica albuginea, and follicular cells of the primordial and primary follicles. The growing and small antral follicles showed strong cytoplasmic and nuclear ERA immunolocalization in the granulosa cells and theca folliculi cells. The large antral (graafian) and pre-ovulatory follicles showed moderate to strong ERA immunolocalization in the granulosa cells, corona radiata cells, cumulus oopherous cells, oocyte, theca interna cells and theca externa cells. The atretic antral follicle showed strong cytoplasmic and negative nuclear ERA immunolocalization in the apoptotic granulosa cells and strong cytoplasmic and nuclear ERA immunolocalization in the proliferated theca interna cells. The endothelial cells of the ovarian blood vessels, the interstitial gland cells and telocytes showed strong cytoplasmic and nuclear ERA immunolocalization. The corpus luteum (CL) during pregnancy till parturition showed moderate to strong ERA immunolocalization in the large lutein cells, small lutein cells and luteal endothelial cells. The regressed CL in the rabbit ovary 10 days of lactation showed weak ERA immunolocalization in the regressed large lutein cells and moderate cytoplasmic and negative nuclear ERA immunolocalization in the small lutein cells. Interestingly, the rabbit ovary during lactation showed abundant interstitial gland with strong ERA immunolocalization in the interstitial gland cells. This work highlights the role of ERA in the ovulation, folliculogenesis, lutenization and luteal regression in the rabbit during pregnancy and lactation which contribute to enhancing this animal’s reproductive success.

## Background

The rabbit is one of the most extensively distributed animals and is frequently utilized for both economical and experimental purposes. In recent times, they have also gained popularity as pets^1^. In addition to being easy to care and inexpensive to feed, and given that rabbit meat has a high nutritional value as it low in fat, cholesterol, and high in protein, raising rabbits may help poor nations overcome their meat scarcity^2^. Among other breeds, New Zealand white rabbit strains are commonly used for research purposes. These strains have fewer health problems and are naturally less aggressive than other breeds^3^. Pregnancy is a crucial time for animals since it causes numerous physiological changes, such as hormone changes, this changes in rabbits do not receive much attention^2^. Rabbit is one of the few animal species that ovulates after mating, resulting in a distinct pregnancy and embryonic age (measured in hours or days following coitus). Rabbits have a short reproductive cycle and thirty-one days make up a pregnancy. For these reasons, rabbits are used as a model of human reproductive health^3^.

Estrogens are the primary female sex hormones and are responsible for regulating several ovarian activities. E2 is synthesized and is present in highest concentrations in the ovaries. The primary mediators of E2 signaling pathways are estrogen receptors (ERs). There are two main types of ERs; the ERA (ERα) and estrogen receptors beta (ERB, ERβ)^4^. Estradiol-17β, the main E2, is produced via androgen aromatization, a process that theca and granulosa cells work together to carry out. Theca cells produce LH-stimulated androgens, which cross the basement membrane to the nearby granulosa cells where aromatase under the influence of FSH, converts them to E2^5,6^.

ERs are transcription factors known to be involved in the regulation of many complex physiological processes in mammals. They are expressed primarily in the reproductive tract of all vertebrates’ females, thus indicating important and conserved functions in female reproductive success. Activation of these nuclear receptors confers sexual differentiation, reproductive function, bone growth and memory storage^7^. They are key receptors to maintain ovarian granulosa cell differentiation, follicle and oocyte growth and development, and ovulation function^8^. ERA is located in different regions of the chromosomes across different animals and commonly expressed in ovarian tissues, including granulosa cells, theca cells, and stromal cells^9,10^. Our previous research^11^ elucidates the role of ERA in the ovarian functions (folliculogenesis, ovulation, lutenization and luteolysis) in rabbit after ovulation induction by HCG.

Therefore, the aim of the current work was to examine the immunolocalization of ERA on the rabbit ovary during pregnancy, postpartum and during lactation. In addition this work helps to understand the regulatory mechanisms of ERA that control folliculogenesis, ovulation, luteinization and luteolysis in the rabbit.

## Martials and methods

### Ethical approval

The experimental protocol was approved by the Local Ethical Committee and by the Institutional Review Board of Molecular Biology Research and studies Institute, Assiut University (IORG0010947-2025) and was carried out in accordance with relevant guidelines and regulations. This research was done in compliance with the ARRIVE guidelines and regulations (https://arrive guidelines.org). All national and institutional guidelines for animal care and use have been followed throughout the study procedures.

### Animals

Eighteen adult healthy sexually mature New Zealand white rabbit does, with a mean weight of 3 ± 0.2 kg were obtained from the animal house, faculty of medicine, Assiut University. The rabbits divided into six groups and lived in separate cages with regulated and constant lighting (12 h), temperature (22–25 °C), ventilation, and humidity. Fertile New Zealand white bucks mated the does. We refer to the day of mating as Day 0. Abdominal palpation can reveal pregnancy 7–10 days after mating. The experiment was done in the animal house, faculty of medicine, Assiut University.

### Samples

Right and left ovaries were collected immediately after slaughtering at 12 h (3 animals), 3 days (3 animals), 7 days (3 animals), 14 days (4 animals) post-mating, at parturition (3 animals) and 10 days lactation (2 animals). The slaughter was carried out by an experienced vet by cutting the jugular vein with a sharp knife. The animals were guaranteed to die before they could be processed any further or sampled. Ovaries were immediately fixed in Bouin’s fluid.

### ERA immunohistochemistry

Immunohistochemical detection of ERA was performed according to^12,13^ and company instruction by using ER (Clone SP1) and an Ultravision Detection System (Anti-Polyvalent, HRP/DAB; Thermo Fisher Scientific, USA) as follow:

1. The ovaries were prepared for paraffin embedding following appropriate fixation.
2. Sections of 3–5 μm were prepared from paraffin blocks, and then they deparaffinized in xylene and rehydrated in descending grades of ethanol (100%, 100%, 95%, 80% and 70%).
3. Sections were given three 5-min washes in PBS (pH 7.4).
4. Slides were treated in 3% hydrogen peroxide for 10 min at room temperature, and then washed in PBS (pH 7.4) to inhibit endogenous peroxidase activities.
5. The slides were heated to almost boiling (95–98 °C) in a water bath for 20 min, cooled for 20 min at room temperature, and then rinsed in PBS in order to retrieve the antigen. This was done using 10 mM sodium citrate buffer (pH6.0).
6. Sections were covered with Ultra V block for five minutes at room temperature in order to suppress the nonspecific background.
7. Sections were treated for 30 min at room temperature with rabbit monoclonal antibody (1: 300) (Cat.#RM-9101S0, Thermo Fisher Scientific, USA).
8. Sections were then washed with PBS at pH 7.4.
9. Sections were treated with a biotinylated secondary antibody [TP-015-BN, Anti-Mouse IgG (H + L), Anti-Rabbit IgG (H + L), 1:300, Thermo Fisher Scientific, USA] at room temperature for 10 min in order to detect the primary antibody.
10. This was followed by several washes in PBS (pH 7.4).
11. Sections were treated with streptavidin-peroxidase complex (Thermo Fisher Scientific, USA) for 10 min at room temperature followed by washing in PBS.
12. The bound antibodies were seen by incubating them for 5 to 15 min at room temperature in a solution that contained one drop of DAB (diaminobenzidine) Plus chromogen and two milliliters of DAB Plus substrate.
13. Harris hematoxylin was used as a counterstain for the sections. The tissues were dehydrated and cover slid with DPX after being cleaned in distilled water.
14. An OLYMPUS BX51 microscope was used to analyze the sections, and an OLYMPUSDP72 camera was used to take pictures.
15. The color of the nucleus and cytoplasm was used to determine the intensity of the immunostaining as follow"
  Dark brown----------------strong immunostaining
  Brown------------------------moderate immunostaining
  Light brown-------------weak immunostaining
  No colour-----------------negative immunostaining.
16. Control negative for ERA immunolocalization in the rabbit ovary was done by omitted the primary antibody to measure background levels. While positive controls such as tissues or cells known to express ERA (as ovary in our study) validate staining patterns and intensity. The immunohistochemistry assay’s sensitivity and specificity are validated by using both kinds of controls.

## Results

**Fig. 1:**
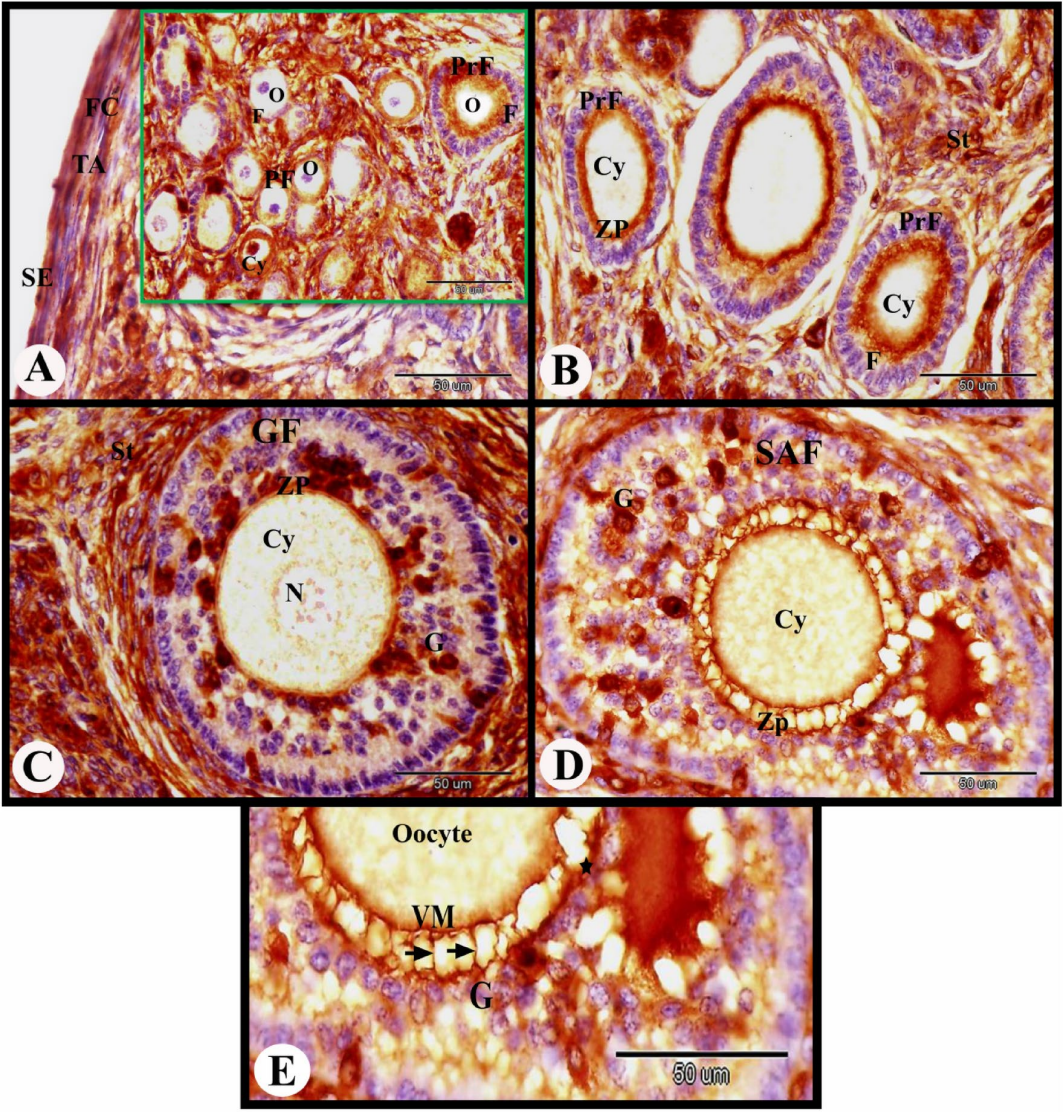
Photomicrograph showing ERA immunolocalization in the rabbit ovary 12 h after mating. (**A**) Showing moderate cytoplasmic ERA immunolocalization in the ovarian surface epithelial cells (SE), and fibroblast cells (FC) of tunica albuginea (TA), moderate cytoplasmic (Cy) and negative nuclear ERA immunolocalization in the flattened follicular cells (F) of the primordial follicles (PF), moderate cytoplasmic and negative nuclear ERA immunolocalization in the cuboidal follicular cells (F) of the primary follicles (PrF), and negative cytoplasmic and nuclear ERA immunolocalization in the oocytes (O) of the primordial and primary follicles. (**B**) Showing moderate cytoplasmic and negative nuclear ERA immunolocalization in the cuboidal follicular cells (F) of the primary follicles (PrF), negative cytoplasmic (Cy) and nuclear ERA immunolocalization in the oocytes of the primary follicles, moderate cytoplasmic and negative nuclear ERA immunolocalization in the stroma cells (St), strong ERA immunolocalization in the zona pellucida (ZP) of the primary follicles. (**C**) Growing follicles (GF) showing weak cytoplasmic (Cy) and negative nuclear (N) ERA immunolocalization in the oocytes, strong ERA immunolocalization in the zona pellucida (ZP) and strong cytoplasmic and nuclear ERA immunolocalization in the granulosa cells (G). Note the moderate cytoplasmic and negative nuclear ERA immunolocalization in the stroma cells (St) surrounded the growing follicle. (**D**) Small antral follicles (SAF) showing strong cytoplasmic and nuclear ERA immunolocalization in the granulosa cells (G), moderate cytoplasmic (Cy) ERA immunolocalization in the oocytes, strong ERA immunolocalization in the zona pellucida (ZP). (**E**) Showing strong ERA immunolocalization in the thin vitelline membrane (VM), zona pellucida (★) and thin cytoplasmic process of granulosa cells (G). Original magnification; A–F × 400, scale bar = 50 µm.

**Fig. 2:**
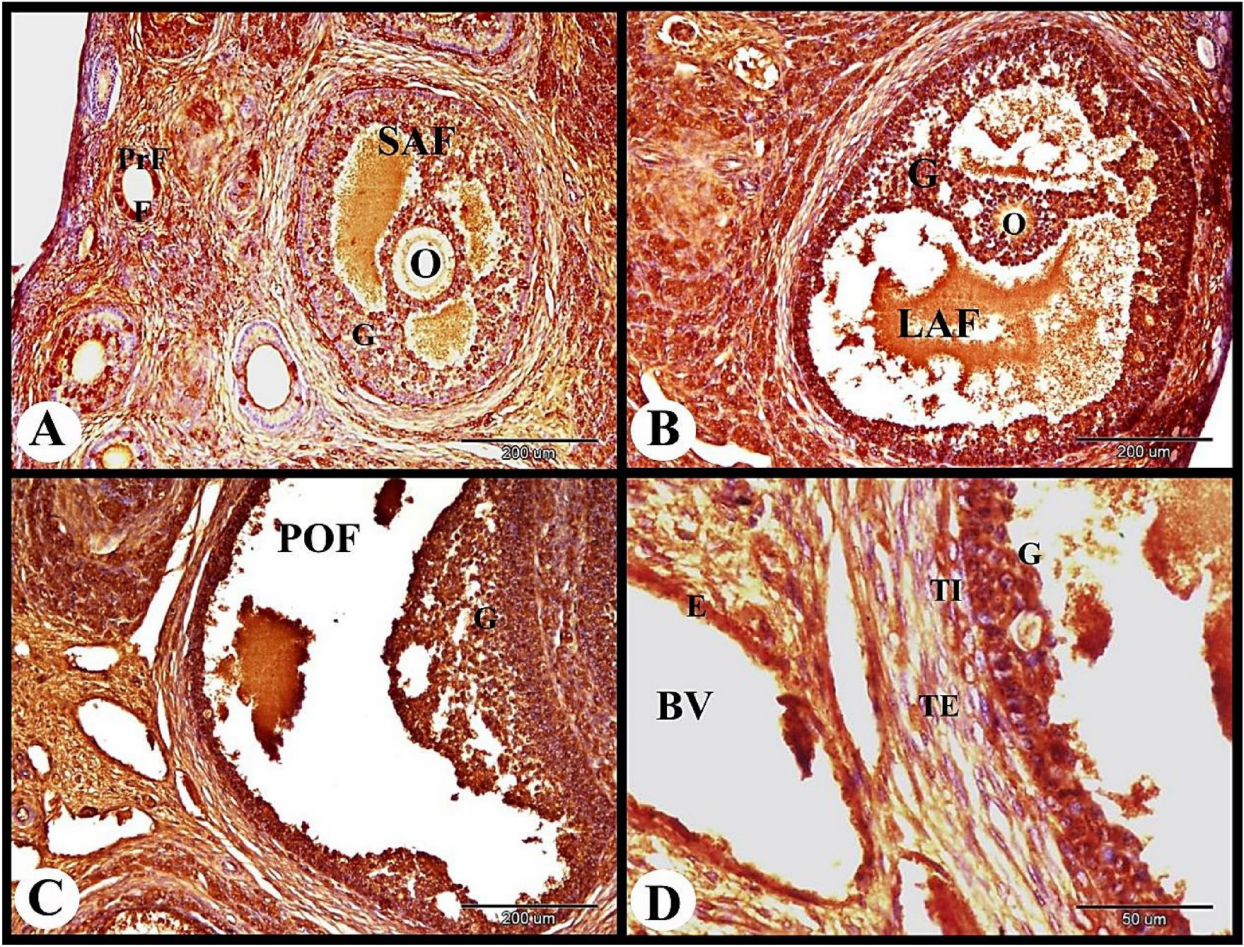
Photomicrograph showing ERA immunolocalization in the rabbit ovary 12 h after mating. (**A**) Small antral follicle (SAF) showing strong cytoplasmic and nuclear ERA immunolocalization in the granulosa cells (G), moderate cytoplasmic ERA immunolocalization in the oocyte and moderate ERA immunolocalization in the cuboidal follicular cells (F) of the primary follicles (PrF). (**B**) Large antral follicle (LAF) showing strong cytoplasmic and nuclear ERA immunolocalization in the granulosa cells (G), negative ERA immunolocalization in the oocyte. (**C**) Showing pre-ovulatory follicle (POF) with strong cytoplasmic and nuclear ERA immunolocalization in the granulosa cells (G). (**D**) Showing strong cytoplasmic and nuclear ERA immunolocalization in the granulosa cells (G), strong cytoplasmic and moderate nuclear ERA immunolocalization in the theca interna cells (TI) and theca externa cells (TE) of the pre-ovulatory follicle. Note strong cytoplasmic and nuclear ERA immunolocalization in the endothelial cells (**E**) of blood vessels (BV). Original magnification; A–C × 100, scale bar = 200 µm, D × 400, scale bar = 50 µm.

In the present study, the cellular distribution of ERA in the rabbit ovary 12 h after mating revealed moderate cytoplasmic ERA immunolocalization in the ovarian surface epithelial cells, fibroblast cells of tunica albuginea, flattened follicular cells of the primordial follicles, and cuboidal follicular cells of the primary follicles. While in the oocytes of the primordial and primary follicles negative cytoplasmic and nuclear ERA immunolocalization was observed (Fig. 1A). Ovarian stroma cells showed moderate cytoplasmic and negative nuclear ERA immunolocalization while the zona pellucida of the primary follicles showed strong ERA immunolocalization (Fig. 1B). The growing follicles showed strong cytoplasmic and nuclear ERA immunolocalization in the granulosa cells, weak cytoplasmic and negative nuclear ERA immunolocalization in the oocytes and strong ERA immunolocalization in the zona pellucida (Fig. 1C). The small antral follicles showed strong cytoplasmic and nuclear ERA immunolocalization in the granulosa cells, moderate cytoplasmic ERA immunolocalization in the oocytes, and strong ERA immunolocalization in the zona pellucida (Fig. 1D). We observed strong ERA immunolocalization in the thin vitelline membrane, zona pellucida and thin cytoplasmic process of granulosa cells in the small antral follicles (Figs. 1E and 2A).

The large antral follicle showed strong cytoplasmic and nuclear ERA immunolocalization in the granulosa cells, and negative ERA immunolocalization in the oocyte (Fig. 2B). The pre-ovulatory follicles revealed strong cytoplasmic and nuclear ERA immunolocalization in the granulosa cells, strong cytoplasmic and moderate nuclear ERA immunolocalization in the theca interna cells and theca externa cells. The ovarian medulla showed strong cytoplasmic and nuclear ERA immunolocalization in the endothelial cells of blood vessels (Fig. 2C,D).

**Fig. 3:**
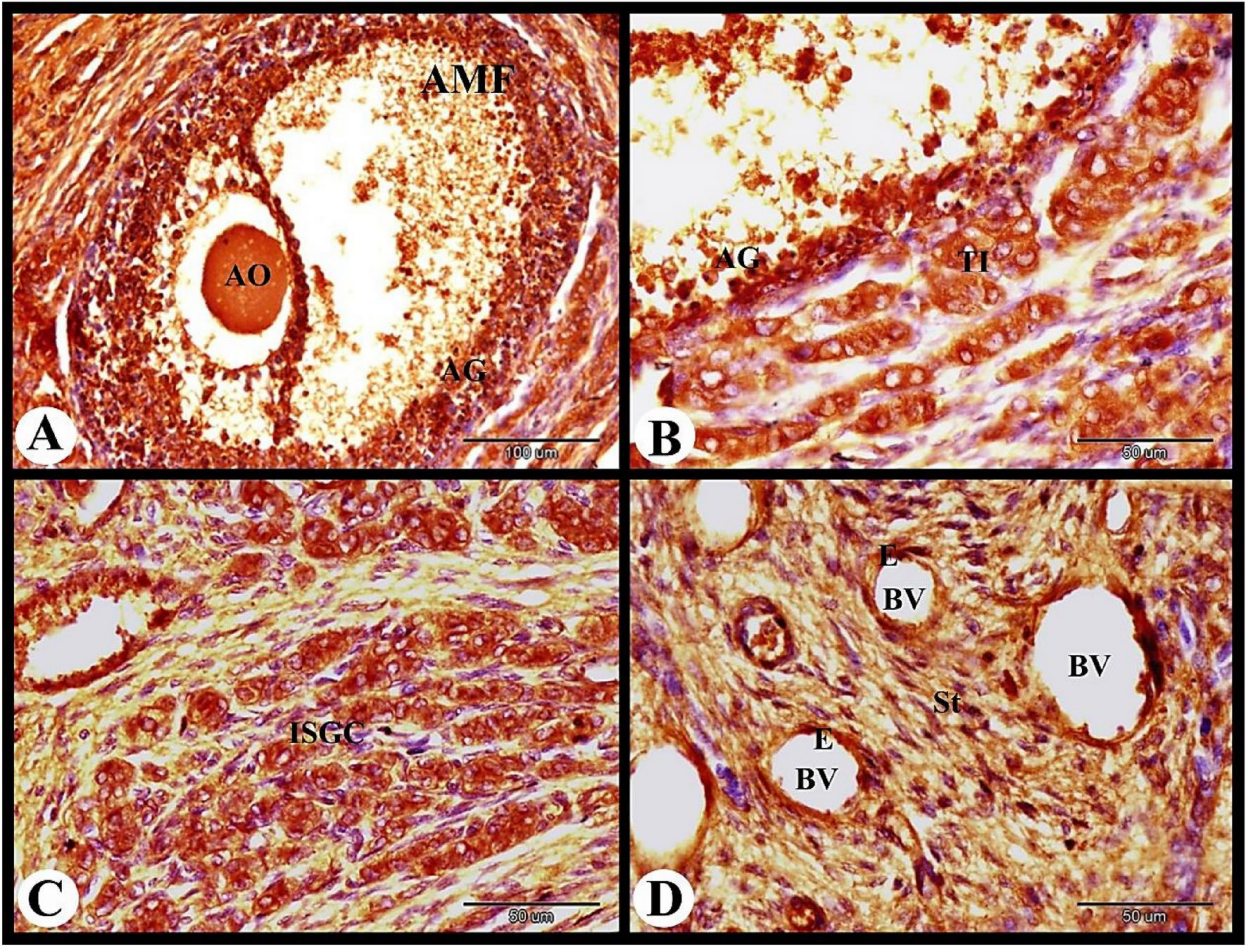
Photomicrograph showing ERA immunolocalization in the rabbit ovary 12 h after mating. (**A**): Showing strong cytoplasmic and negative nuclear ERA immunolocalization in the apoptotic granulosa cells (AG) and strong cytoplasmic ERA immunolocalization in the apoptotic oocyte (AO) of the atretic mature follicle (AMF). (**B**): Higher magnification of the wall of atretic follicle showing strong cytoplasmic and negative nuclear ERA immunolocalization in the sloughed apoptotic granulosa cells (AG) and strong cytoplasmic and nuclear ERA immunolocalization in the proliferated theca interna cells (TI). (**C**): Showing strong cytoplasmic and nuclear ERA immunolocalization in the interstitial gland cells (ISGC). (**D**): Showing strong cytoplasmic and nuclear ERA immunolocalization in the endothelial cells (**E**) of the ovarian blood vessels (BV) and moderate cytoplasmic and nuclear ERA immunolocalization in the stroma cells (St). Original magnification; A × 200, scale bar = 100 µm, B–D × 400, scale bar = 50 µm.

The atretic mature graafian follicle at 12 h post mating showed strong cytoplasmic and negative nuclear ERA immunolocalization in the apoptotic granulosa cells and strong cytoplasmic ERA immunolocalization in the apoptotic oocyte (Fig. 3A). In addition we observed strong cytoplasmic and negative nuclear ERA immunolocalization in the sloughed apoptotic granulosa cells and strong cytoplasmic and nuclear ERA immunolocalization in the proliferated theca interna cells (Fig. 3B). The interstitial gland cells showed strong cytoplasmic and nuclear ERA immunolocalization ((Fig. 3C). As well as the ovarian medulla showed strong cytoplasmic and nuclear ERA immunolocalization in the endothelial cells of the medullary blood vessels and moderate cytoplasmic and nuclear ERA immunolocalization in the medullary stromal cells (Fig. 3D).

**Fig. 4:**
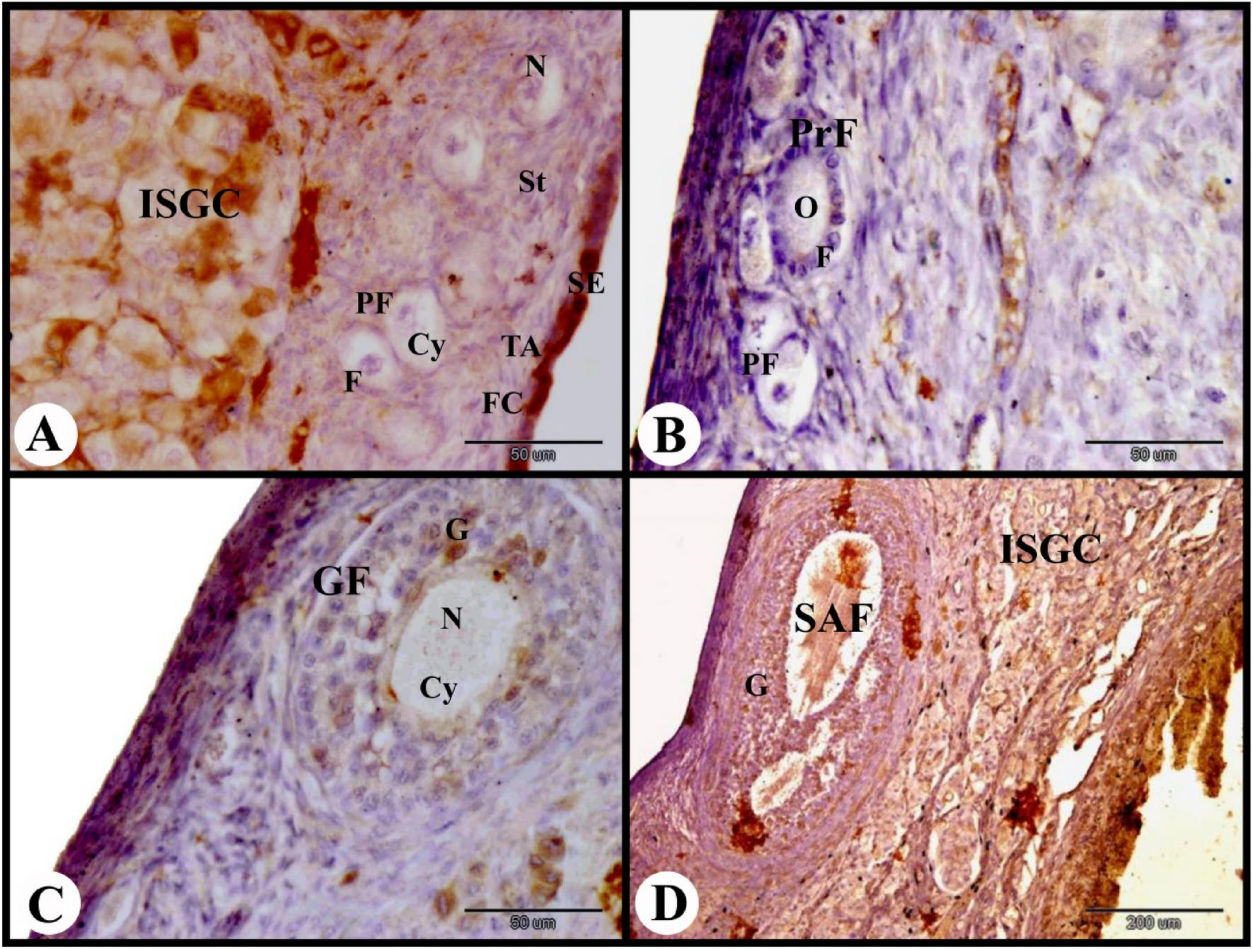
Photomicrograph showing ERA immunolocalization in the rabbit ovary 3 days of pregnancy. (**A**): Showing strong cytoplasmic and nuclear ERA immunolocalization in the ovarian surface epithelial cells (SE), weak cytoplasmic and negative nuclear ERA immunolocalization in the fibroblast cells (FC) in the tunica albuginea (TA), weak to negative cytoplasmic and nuclear ERA immunolocalization in the flattened follicular cells (F) of the primordial follicles (PF), negative cytoplasmic (Cy) and nuclear (N) ERA immunolocalization in the oocytes of the primordial follicles (PF), weak cytoplasmic and negative nuclear ERA immunolocalization in the stroma cells (St) and moderate cytoplasmic and nuclear ERA immunolocalization in the interstitial gland cells (ISGC). (**B**): Showing weak to negative cytoplasmic and nuclear ERA immunolocalization in the cuboidal follicular cells (F) of the primary follicles (PrF), and negative cytoplasmic and nuclear ERA immunolocalization in the oocyte (O) of the primary follicles (PrF). Note negative ERA immunolocalization in the primordial follicles (PF). (**C**): Showing weak to moderate cytoplasmic and nuclear ERA immunolocalization in the granulosa cells (G) of the growing follicle (GF), and negative cytoplasmic (Cy) and nuclear (N) ERA immunolocalization in the oocyte of the growing follicle (GF). (**D**): Showing moderate cytoplasmic and nuclear ERA immunolocalization in the granulosa cells (G) of the small antral follicle (SAF) and in the interstitial gland cells (ISGC). Original magnification; A–C × 400, scale bar = 50 µm, D × 100, scale bar = 200 µm.

**Fig. 5:**
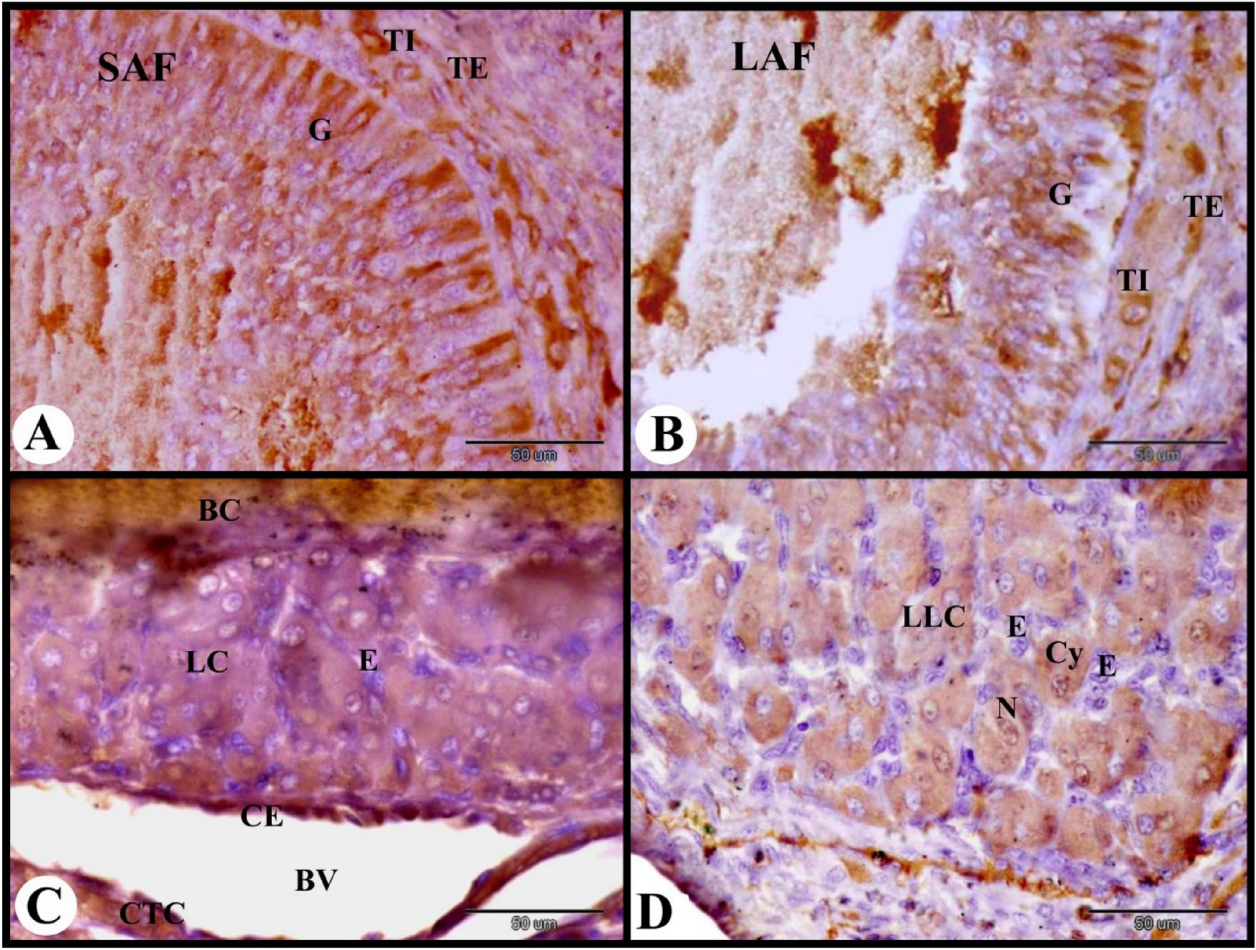
Photomicrograph showing ERA immunolocalization in the rabbit ovary 3 days of pregnancy. (**A**): Showing moderate cytoplasmic ERA immunolocalization in the granulosa cells (G) and moderate cytoplasmic and nuclear ERA immunolocalization in the theca interna (TI) and theca externa cells (TE) of the small antral follicle (SAF). (**B**): Showing moderate cytoplasmic and weak nuclear ERA immunolocalization in the granulosa cells (G), the theca interna cells (TI) and theca externa cells (TE) of the large antral follicle (LAF). (**C**): Showing moderate cytoplasmic and nuclear ERA immunolocalization in the lutein cells (LC), negative cytoplasmic and nuclear ERA immunolocalization in the endothelial cells (E), and strong cytoplasmic and nuclear ERA immunolocalization in the capsular endothelial cells (CE) of the blood vessels (BV) of the connective tissue capsule (CTC) of the developing CL. Note the central blood clot (BC). (**D**): Showing moderate cytoplasmic (Cy) and nuclear (N) ERA immunolocalization in the large lutein cells (LLC), and negative cytoplasmic and nuclear ERA immunolocalization in the endothelial cells (E) of the developing CL. Original magnification; A–D × 400, scale bar = 50 µm.

ERA immunolocalization in the rabbit ovary 3 days of pregnancy showed strong cytoplasmic and nuclear ERA immunolocalization in the ovarian surface epithelial cells and weak cytoplasmic and negative nuclear ERA immunolocalization in the fibroblast cells of the tunica albuginea. The primordial follicles showed weak to negative cytoplasmic and nuclear ERA immunolocalization in the flattened follicular cells and negative cytoplasmic and nuclear ERA immunolocalization in the oocytes. While the stroma cells showed weak cytoplasmic and negative nuclear ERA immunolocalization and the interstitial gland cells showed moderate cytoplasmic and nuclear ERA immunolocalization (Fig. 4A). The primary follicles showed weak to negative cytoplasmic and nuclear ERA immunolocalization in the cuboidal follicular cells and negative cytoplasmic and nuclear ERA immunolocalization in the oocyte (Fig. 4B). The growing follicle showed weak to moderate cytoplasmic and nuclear ERA immunolocalization in the granulosa cells and negative cytoplasmic and nuclear ERA immunolocalization in the oocyte (Fig. 4C). The small antral follicle showed moderate cytoplasmic and nuclear ERA immunolocalization in the granulosa cells, theca interna cells and theca externa cells (Figs. 4D and 5A).

The large antral follicle at 3 days of pregnancy showed moderate cytoplasmic and weak nuclear ERA immunolocalization in the granulosa cells, theca interna cells and theca externa cells (Fig. 5B). The developing CL at this stage of pregnancy showed moderate cytoplasmic and nuclear ERA immunolocalization in the large lutein cells, negative cytoplasmic and nuclear ERA immunolocalization in the endothelial cells of blood capillaries, and strong cytoplasmic and nuclear ERA immunolocalization in the capsular endothelial cells (Fig. 5C,D).

**Fig. 6:**
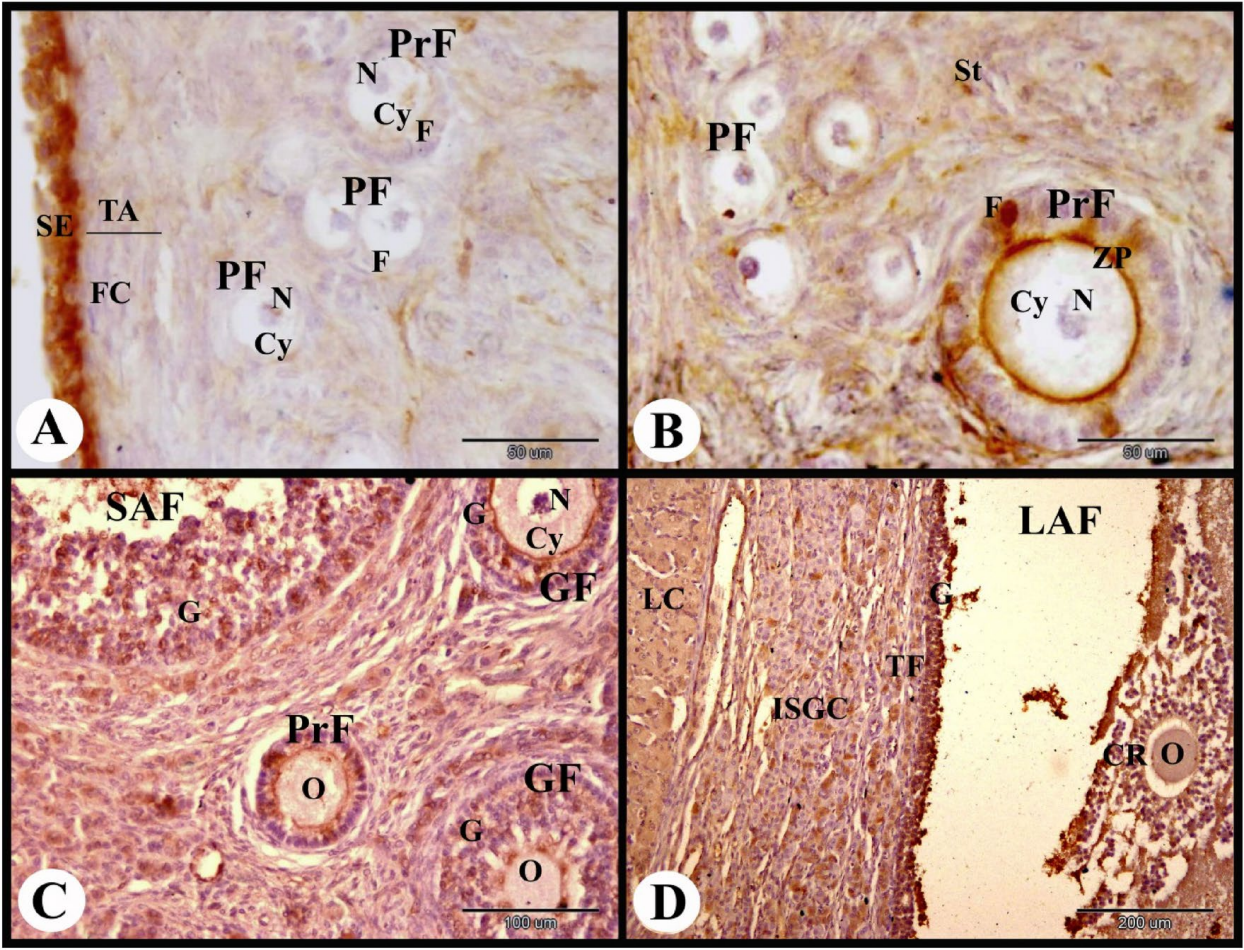
Photomicrograph showing ERA immunolocalization in the rabbit ovary 7 days of pregnancy. (**A**): Showing moderate cytoplasmic and nuclear ERA immunolocalization in the ovarian surface epithelial cells (SE), weak cytoplasmic and negative nuclear ERA immunolocalization in the fibroblast cells (FC) in the tunica albuginea (TA), weak to negative cytoplasmic and nuclear ERA immunolocalization in the flattened follicular cells (F) of the primordial follicles (PF), negative cytoplasmic (Cy) and nuclear (N) ERA immunolocalization in the oocytes of the primordial follicles (PF), and weak cytoplasmic (Cy) and negative nuclear (N) ERA immunolocalization in the cuboidal follicular cells (F) and oocyte of the early primary follicles (PrF). (**B**): Showing moderate cytoplasmic and nuclear ERA immunolocalization in the cuboidal follicular cells (F) of the late primary follicles (PrF), weak cytoplasmic and negative nuclear ERA immunolocalization in the oocyte (O) of the late primary follicles (PrF), and weak cytoplasmic and negative nuclear ERA immunolocalization in the stroma cells (St). Note strong ERA immunolocalization in the zona pellucida (ZP) and weak ERA immunolocalization in the primordial follicles (PF). (**C**): Showing moderate cytoplasmic and nuclear ERA immunolocalization in the granulosa cells (G) of the growing follicle (GF) and small antral follicle (SAF), and negative cytoplasmic (Cy) and nuclear (N) ERA immunolocalization in the oocyte (O) of the growing follicle (GF). Note the weak to moderate ERA immunolocalization in the follicular cells and oocyte (O) of the primary follicles (PrF). (**D**): Showing moderate ERA immunolocalization in the granulosa cells (G), theca folliculi cells (TF), oocyte (O), and corona radiata cells (CR) of the large antral follicle (LAF) and in the interstitial gland cells (ISGC) and luteal cells (LC). Original magnification; A, B × 400, scale bar = 50 µm, C × 200, scale bar = 100 µm, D × 100, scale bar = 200 µm.

ERA immunolocalization in the rabbit ovary at 7 days of pregnancy showed moderate cytoplasmic and nuclear ERA immunolocalization in the ovarian surface epithelial cells, weak cytoplasmic and negative nuclear ERA immunolocalization in the fibroblast cells of the tunica albuginea. The primordial follicles showed weak to negative cytoplasmic and nuclear ERA immunolocalization in the flattened follicular cells and negative cytoplasmic and nuclear ERA immunolocalization in the oocytes. The early primary follicles showed weak cytoplasmic and negative nuclear ERA immunolocalization in the cuboidal follicular cells and oocyte (Fig. 6A). The late primary follicles showed moderate cytoplasmic and nuclear ERA immunolocalization in the cuboidal follicular cells and weak cytoplasmic and negative nuclear ERA immunolocalization in the oocyte. The stromal cells showed weak cytoplasmic and negative nuclear ERA immunolocalization while the zona pellucida showed strong ERA immunolocalization (Fig. 6B). The growing and small antral follicles showed moderate cytoplasmic and nuclear ERA immunolocalization in the granulosa cells while the oocytes of the growing follicle showed negative cytoplasmic and nuclear ERA immunolocalization (Fig. 6C). The large antral follicle showed moderate ERA immunolocalization in the granulosa cells, theca folliculi cells, corona radiata cells and oocyte. The interstitial gland cells and luteal cells also showed moderate ERA immunolocalization (Fig. 6D).

**Fig. 7:**
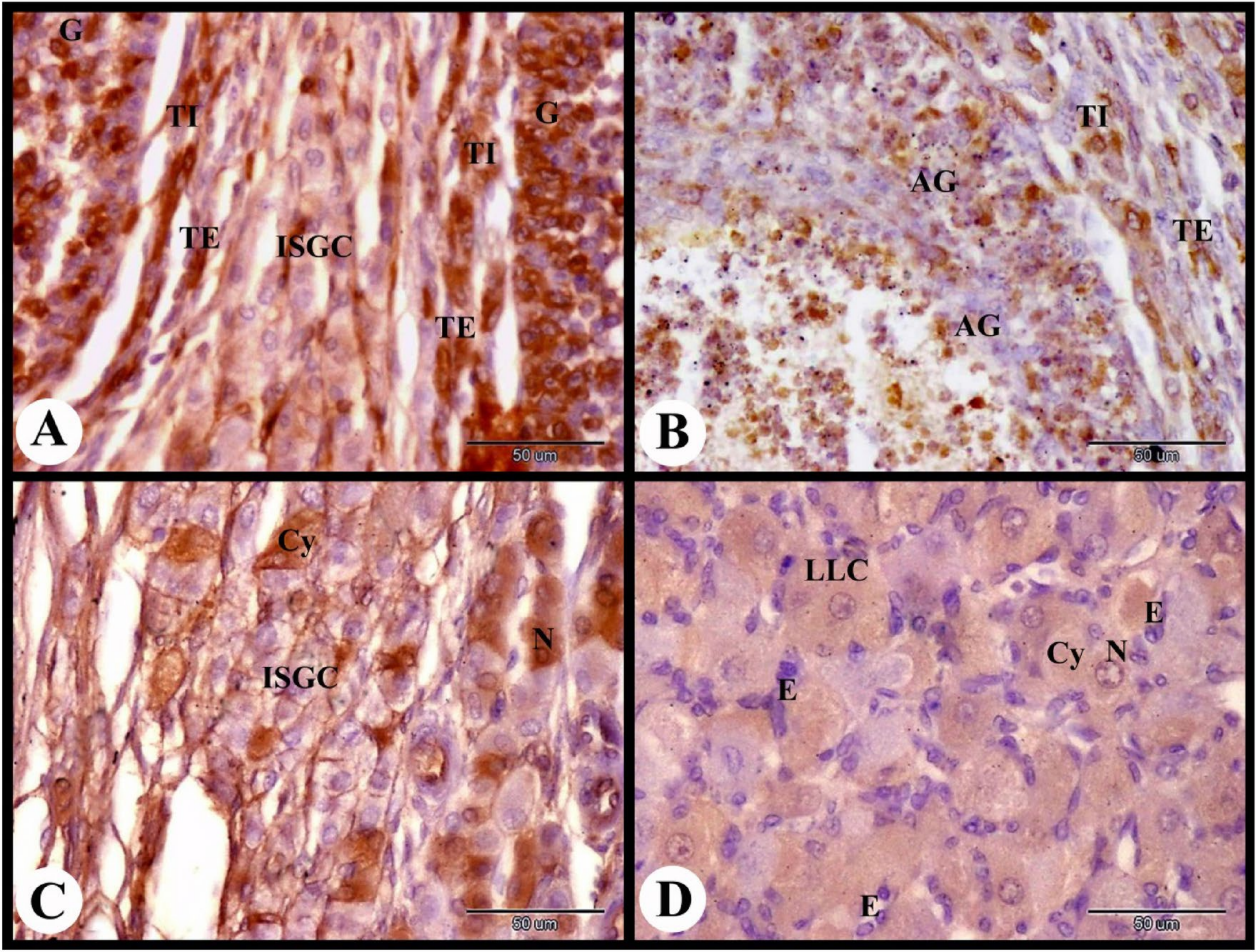
Photomicrograph showing ERA immunolocalization in the rabbit ovary 7 days of pregnancy. (**A**): Showing moderate cytoplasmic and nuclear ERA immunolocalization in the granulosa cells (G), theca interna cells (TI) and theca externa cells (TE) of the large antral follicle and in the interstitial gland cells (ISGC). (**B**): Showing negative cytoplasmic and nuclear ERA immunolocalization in the apoptotic granulosa cells (AG) and moderate cytoplasmic and nuclear ERA immunolocalization in the theca interna cells (TI) and theca externa cells (TE) of the atretic antral follicle. (**C**): Showing moderate cytoplasmic (Cy) and nuclear (N) ERA immunolocalization in the interstitial gland cells (ISGC). (**D**): Showing moderate cytoplasmic (Cy) and nuclear (N) ERA immunolocalization in the large lutein cells (LLC) and moderate cytoplasmic and negative nuclear ERA immunolocalization in the luteal endothelial cells (E) of CL. Original magnification; A–D × 400, scale bar = 50 µm.

At this stage of pregnancy the large antral follicle showed moderate cytoplasmic and nuclear ERA immunolocalization in the granulosa cells, theca interna cells and theca externa cells (Fig. 7A). Whereas the atretic antral follicle showed negative cytoplasmic and nuclear ERA immunolocalization in the apoptotic granulosa cells and moderate cytoplasmic and nuclear ERA immunolocalization in the theca interna cells and theca externa cells (Fig. 7B). The interstitial gland cells showed moderate cytoplasmic and nuclear ERA immunolocalization (Fig. 7C). The large lutein cells showed moderate cytoplasmic and nuclear ERA immunolocalization while the luteal endothelial cells showed moderate cytoplasmic and negative nuclear ERA immunolocalization (Fig. 7D).

**Fig. 8:**
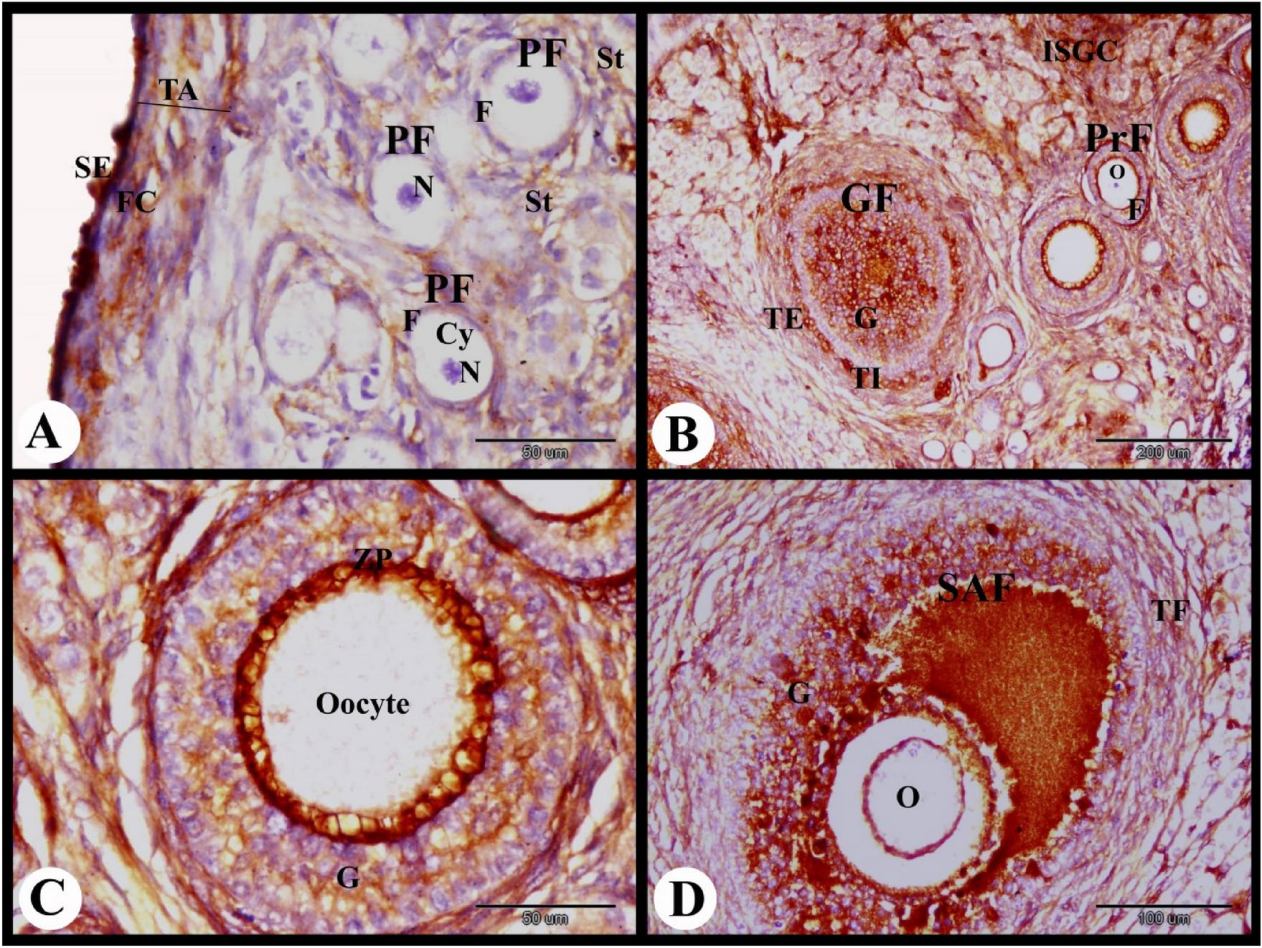
Photomicrograph showing ERA immunolocalization in the rabbit ovary 14 days of pregnancy. (**A**): Showing strong cytoplasmic and nuclear ERA immunolocalization in the ovarian surface epithelial cells (SE), moderate cytoplasmic and negative nuclear ERA immunolocalization in the fibroblast cells (FC) in the tunica albuginea (TA), weak cytoplasmic and negative nuclear ERA immunolocalization in the flattened follicular cells (F) of the primordial follicles (PF) and stroma cells (St), negative cytoplasmic (Cy) and nuclear (N) ERA immunolocalization in the oocytes of the primordial follicles (PF). (**B**): Showing moderate cytoplasmic and negative nuclear ERA immunolocalization in the columnar follicular cells (F) of the primary follicles (PrF), negative ERA immunolocalization in the oocyte (O) of the primary follicles (PrF), and moderate cytoplasmic and nuclear ERA immunolocalization in the interstitial gland cells (ISGC), and strong cytoplasmic and nuclear ERA immunolocalization in the granulosa cells (G) and theca interna cells (TI) and moderate cytoplasmic and nuclear ERA immunolocalization in the theca externa cells (TE) of the growing follicles (GF) (**C**): Showing moderate cytoplasmic and negative nuclear ERA immunolocalization in the granulosa cells (G), of the growing follicles (GF) and negative ERA immunolocalization in the oocyte (O) and strong ERA immunolocalization in the zona pellucida (ZP) of the growing follicle (GF). (**D**): Showing strong cytoplasmic and nuclear ERA immunolocalization in the granulosa cells (G), negative ERA immunolocalization in the oocyte (O) and moderate cytoplasmic and nuclear ERA immunolocalization in the theca folliculi cells (TF) of the small antral follicle (SAF). Original magnification; A × 400, scale bar = 50 µm, B × 100, scale bar = 200 µm, C × 400, scale bar = 50 µm, D × 200, scale bar = 100 µm.

At the middle stage of pregnancy (14 days post mating) the rabbit ovary showed strong cytoplasmic and nuclear ERA immunolocalization in the ovarian surface epithelial cells, moderate cytoplasmic and negative nuclear ERA immunolocalization in the fibroblast cells of the tunica albuginea. The primordial follicles showed weak cytoplasmic and negative nuclear ERA immunolocalization in the flattened follicular cells and negative cytoplasmic and nuclear ERA immunolocalization in the oocytes. The stroma cells showed weak cytoplasmic and negative nuclear ERA immunolocalization (Fig. 8A). The primary follicles showed moderate cytoplasmic and negative nuclear ERA immunolocalization in the columnar follicular cells and negative ERA immunolocalization in the oocyte. The growing follicles showed strong cytoplasmic and nuclear ERA immunolocalization in the granulosa cells and theca interna cells, and moderate cytoplasmic and nuclear ERA immunolocalization in the theca externa cells. Some early growing follicles showed moderate cytoplasmic and negative nuclear ERA immunolocalization in the granulosa cells, negative ERA immunolocalization in the oocyte and strong ERA immunolocalization in the zona pellucida (Fig. 8B,C). The small antral follicle showed strong cytoplasmic and nuclear ERA immunolocalization in the granulosa cells, negative ERA immunolocalization in the oocyte and moderate cytoplasmic and nuclear ERA immunolocalization in the theca folliculi cells (Fig. 8D).

**Fig. 9:**
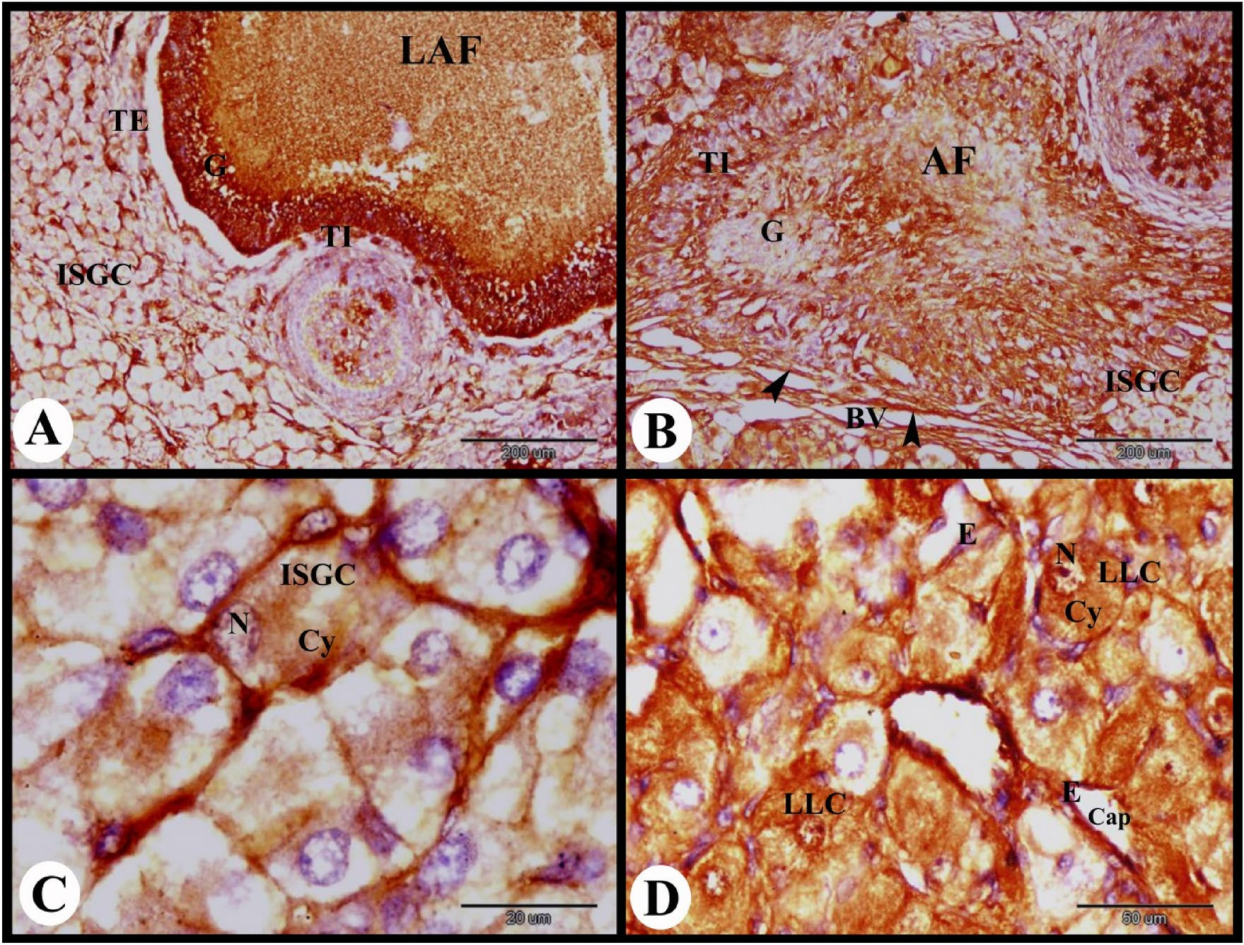
Photomicrograph showing ERA immunolocalization in the rabbit ovary 14 days of pregnancy. (**A**): Showing strong cytoplasmic and nuclear ERA immunolocalization in the granulosa cells (G) and theca interna cells (TI) and moderate cytoplasmic and nuclear ERA immunolocalization in the theca externa cells (TE) of the large antral follicle and moderate ERA immunolocalization in the interstitial gland cells (ISGC). (**B**): Showing negative cytoplasmic and nuclear ERA immunolocalization in the granulosa cells (G) and moderate cytoplasmic and nuclear ERA immunolocalization in the proliferated theca interna cells (TI) of the atretic follicle. Note strong cytoplasmic and nuclear ERA immunolocalization in the endothelial cells (arrowheads) of blood vessels (BV) and moderate ERA immunolocalization in the interstitial gland cells (ISGC). (**C**): Showing moderate cytoplasmic (Cy) and nuclear (N) ERA immunolocalization in the interstitial gland cells (ISGC). (**D**): Showing strong cytoplasmic (Cy) and nuclear (N) ERA immunolocalization in the large lutein cells (LLC) and moderate cytoplasmic and negative nuclear ERA immunolocalization in the endothelial cells (E) of the luteal blood capillaries (Cap) of the CL. Original magnification; A, B × 100, scale bar = 200 µm, C × 1000, scale bar = 20 µm, D × 400, scale bar = 50 µm.

At this stage of gestation the large antral follicle showed strong cytoplasmic and nuclear ERA immunolocalization in the granulosa cells and theca interna cells and moderate cytoplasmic and nuclear ERA immunolocalization in the theca externa cells (Fig. 9A). While the atretic follicle showed negative cytoplasmic and nuclear ERA immunolocalization in the granulosa cells and moderate cytoplasmic and nuclear ERA immunolocalization in the proliferated theca interna cells. As well as the endothelial cells of blood vessels showed strong cytoplasmic and nuclear ERA immunolocalization (Fig. 9B). The interstitial gland cells showed moderate cytoplasmic and nuclear ERA immunolocalization (Fig. 9A–C). The large lutein cells showed strong cytoplasmic and nuclear ERA immunolocalization and the endothelial cells of the luteal blood capillaries showed moderate cytoplasmic and negative nuclear ERA immunolocalization (Fig. 9D).

**Fig. 10:**
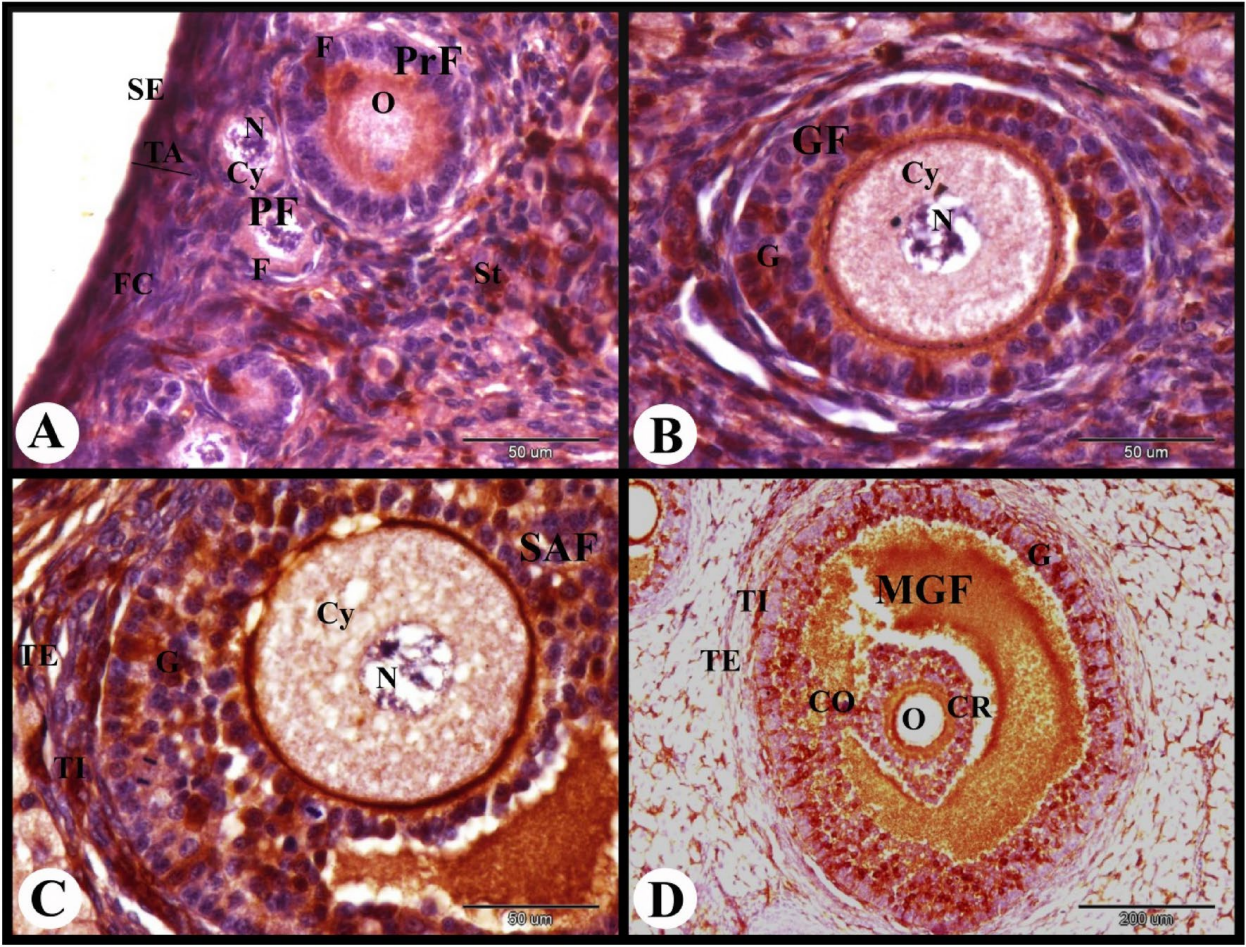
Photomicrograph showing ERA immunolocalization in the parturient rabbit ovary. (**A**): Showing strong cytoplasmic and nuclear ERA immunolocalization in the ovarian surface epithelial cells (SE), moderate cytoplasmic and negative nuclear ERA immunolocalization in the fibroblast cells (FC) in the tunica albuginea (TA), weak cytoplasmic and negative nuclear ERA immunolocalization in the flattened follicular cells (F) of the primordial follicles (PF) and stroma cells (St), negative cytoplasmic (Cy) and nuclear (N) ERA immunolocalization in the oocytes of the primordial follicles (PF), moderate cytoplasmic and negative nuclear ERA immunolocalization in the follicular cells (F) of the primary follicles (PrF), negative ERA immunolocalization in the oocyte (O) of the primary follicles (PrF). (**B**): Showing moderate cytoplasmic and nuclear ERA immunolocalization in the granulosa cells, weak cytoplasmic (Cy) and negative nuclear (N) ERA immunolocalization in the oocyte of the growing follicle (GF). (**C**): Showing moderate cytoplasmic and nuclear ERA immunolocalization in the granulosa cells (G), theca interna cells (TI), theca externa cells (TE), weak cytoplasmic (Cy) and negative nuclear (N) ERA immunolocalization in the oocyte of the small antral follicle (SAF). (**D**): Showing moderate cytoplasmic and nuclear ERA immunolocalization in the granulosa cells (G), corona radiate cells (CR), cumulus oopherous cells (CO), theca interna cells (TI) and theca externa cells (TE) and negative ERA immunolocalization in the oocyte (O) of the mature graafian follicles (MGF). Original magnification; A–C × 400, scale bar = 50 µm, D × 100, scale bar = 200 µm.

ERA immunolocalization in the parturient rabbit ovary showed strong cytoplasmic and nuclear ERA immunolocalization in the ovarian surface epithelial cells, moderate cytoplasmic and negative nuclear ERA immunolocalization in the fibroblast cells of the tunica albuginea. The primordial follicles showed weak cytoplasmic and negative nuclear ERA immunolocalization in the flattened follicular cells and negative cytoplasmic and nuclear ERA immunolocalization in the oocytes. The stroma cells showed weak cytoplasmic and negative nuclear ERA immunolocalization. Whereas the primary follicles showed moderate cytoplasmic and negative nuclear ERA immunolocalization in the follicular cells and negative ERA immunolocalization in the oocyte (Fig. 10A). The growing follicle showed moderate cytoplasmic and nuclear ERA immunolocalization in the granulosa cells and weak cytoplasmic and negative nuclear ERA immunolocalization in the oocyte (Fig. 10B).

**Fig. 11:**
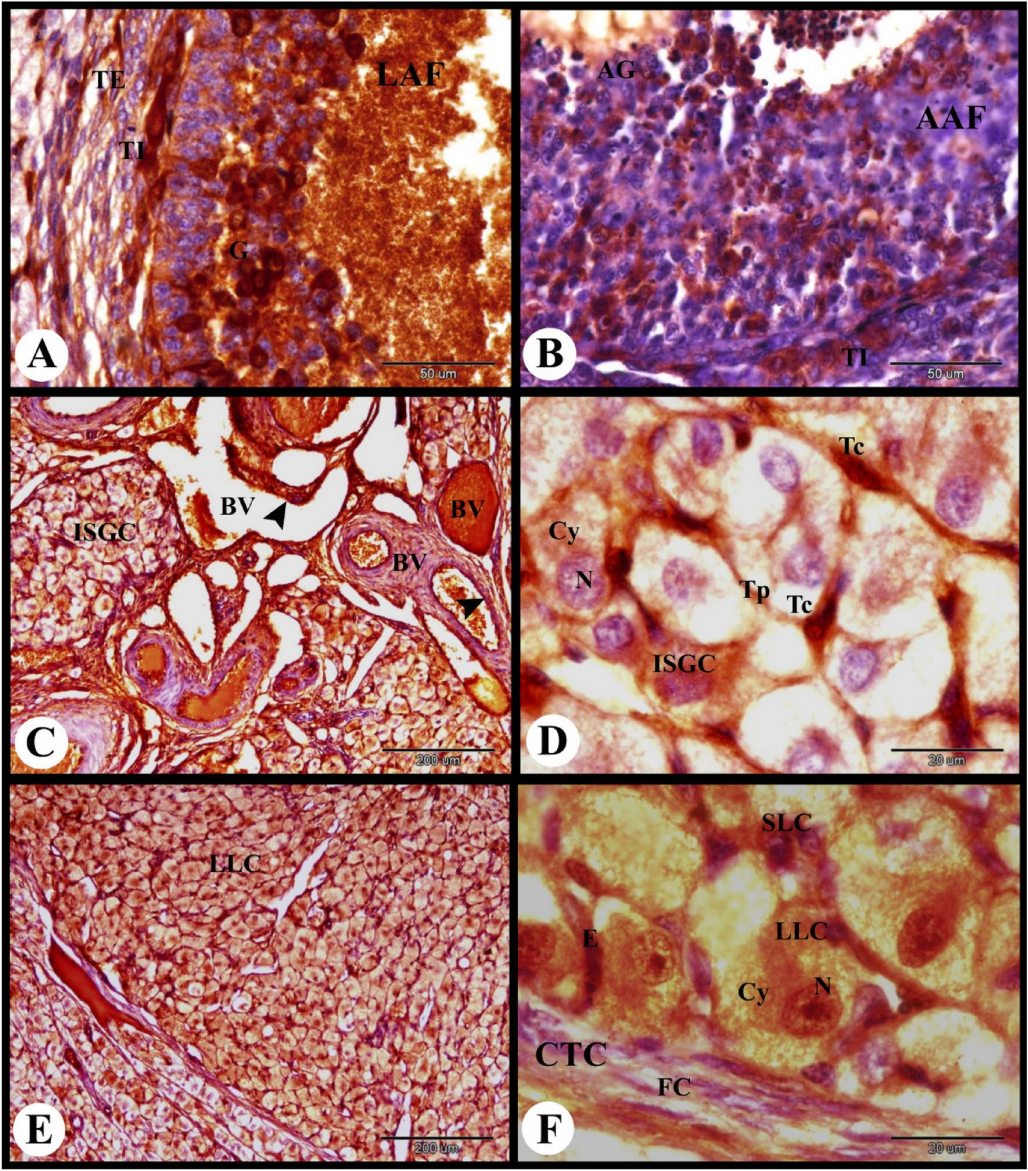
Photomicrograph showing ERA immunolocalization in the parturient rabbit ovary. (**A**): Showing moderate cytoplasmic and nuclear ERA immunolocalization in the granulosa cells (G), theca interna cells (TI) and theca externa cells (TE) of the large antral follicle (LAF). (**B**): Showing negative cytoplasmic and nuclear ERA immunolocalization in the apoptotic granulosa cells (AG) and moderate cytoplasmic and nuclear ERA immunolocalization in the proliferated theca interna cells (TI) of the atretic antral follicle (AAF). (**C**,**D**): Showing moderate cytoplasmic (Cy) and nuclear (N) ERA immunolocalization in the interstitial gland cells (ISGC) and strong cytoplasmic and nuclear ERA immunolocalization in the endothelial cells (arrowheads) of medullary blood vessels (BV). Note strong cytoplasmic and nuclear ERA immunolocalization in the telocytes (Tc) and telopoda (Tp). (**E**,**F**): Showing moderate cytoplasmic (Cy) and nuclear (N) ERA immunolocalization in the large lutein cells (LLC), small lutein cells (SLC) and in the endothelial cells (E) of the CL. Note weak cytoplasmic and nuclear ERA immunolocalization in the fibroblast cells (FC) of the connective tissue capsule (CTC) of CL. Original magnification; A, B × 100, scale bar = 200 µm, C × 1000, scale bar = 20 µm, D × 400, scale bar = 50 µm.

The small antral follicle showed moderate cytoplasmic and nuclear ERA immunolocalization in the granulosa cells, theca interna cells, and theca externa cells and weak cytoplasmic and negative nuclear ERA immunolocalization in the oocyte (Fig. 10C). The mature graafian follicles showed moderate cytoplasmic and nuclear ERA immunolocalization in the granulosa cells, corona radiate cells, cumulus oopherous cells, theca interna cells and theca externa cells while they showed negative ERA immunolocalization in the oocyte (Figs. 10D and 11A).

The atretic antral follicle of the parturient rabbit ovary showed negative cytoplasmic and nuclear ERA immunolocalization in the apoptotic granulosa cells and moderate cytoplasmic and nuclear ERA immunolocalization in the proliferated theca interna cells (Fig. 11B). The interstitial gland cells showed moderate cytoplasmic and nuclear ERA immunolocalization. Telocytes and telopoda in the interstitial glands showed strong cytoplasmic and nuclear ERA immunolocalization. While the endothelial cells of the medullary blood vessels showed strong cytoplasmic and nuclear ERA immunolocalization (Fig. 11C,D). The CL showed moderate cytoplasmic and nuclear ERA immunolocalization in the large lutein cells, small lutein cells and in the endothelial cells. Whereas, weak cytoplasmic and nuclear ERA immunolocalization in the fibroblast cells of the connective tissue capsule of the CL was observed (Fig. 11E,F).

**Fig. 12:**
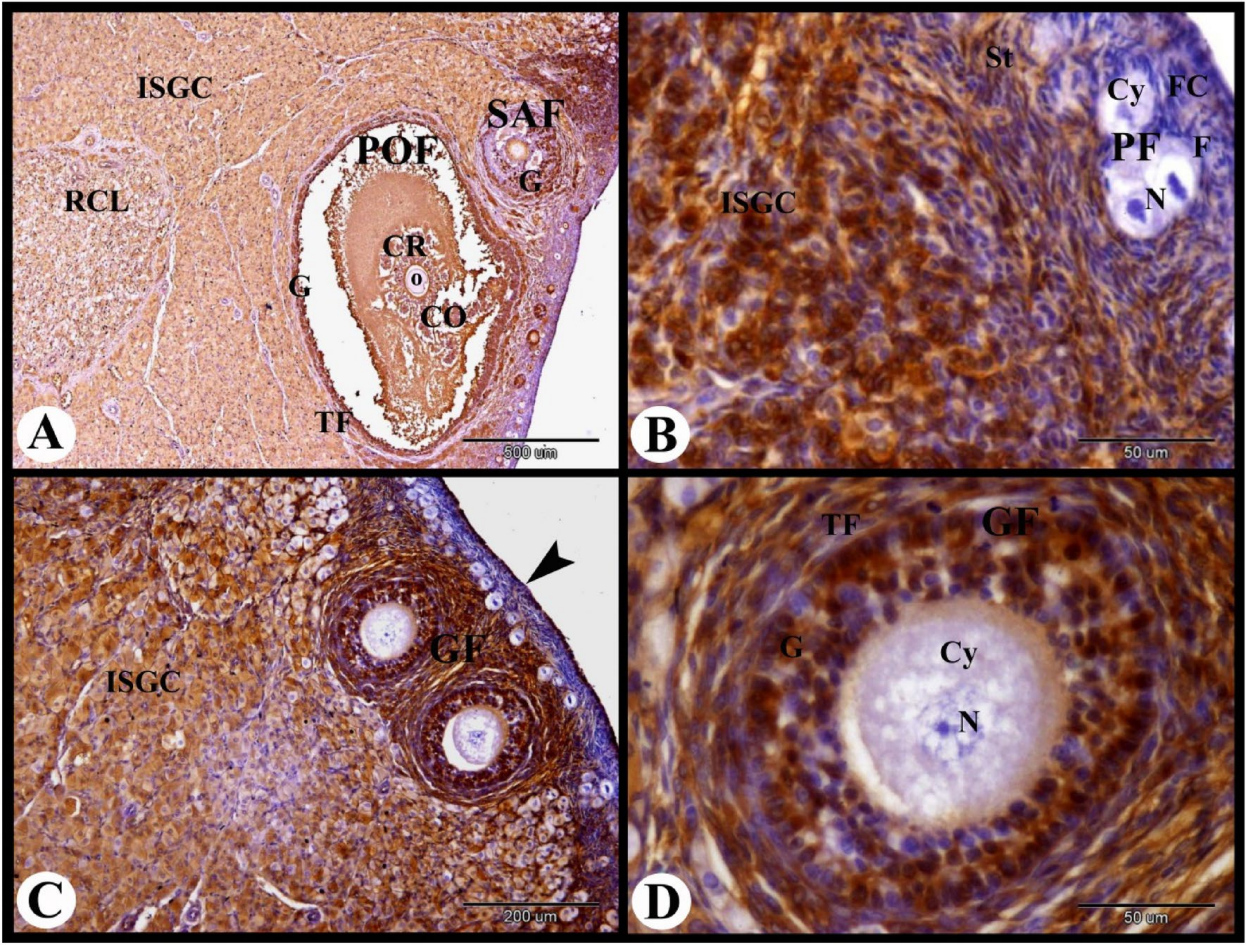
Photomicrograph showing ERA immunolocalization in the rabbit ovary 10 days of lactation. (**A**): Showing strong cytoplasmic and nuclear ERA immunolocalization in the granulosa cells (G), theca folliculi (TF), corona radiate cells (CR), cumulus oopherous cells (CO), and negative ERA immunolocalization in the oocyte (O) of the preovulatory follicles (POF), moderate cytoplasmic and nuclear ERA immunolocalization in the interstitial gland cells (ISGC) and granulosa cells (G) of the small antral follicles (SAF). Note the regressed CL (RCL). (**B**): Showing weak cytoplasmic and negative nuclear ERA immunolocalization in the fibroblast cells (FC) in the tunica albuginea, weak cytoplasmic and negative nuclear ERA immunolocalization in the flattened follicular cells (F) of the primordial follicles (PF), negative cytoplasmic (Cy) and nuclear (N) ERA immunolocalization in the oocytes of the primordial follicles (PF), moderate cytoplasmic and nuclear ERA immunolocalization in the stroma cells (St), and strong cytoplasmic and nuclear ERA immunolocalization in the interstitial gland cells (ISGC). (**C**): Showing strong cytoplasmic and nuclear ERA immunolocalization in the ovarian surface epithelial cells (arrowheads), strong cytoplasmic and nuclear ERA immunolocalization in the granulosa cells (G) of the growing follicles (GF) and in the interstitial gland cells (ISGC). (**D**): Showing strong cytoplasmic and nuclear ERA immunolocalization in the granulosa cells (G), and moderate cytoplasmic and nuclear ERA immunolocalization in the theca folliculi cells (TF) of the growing follicles (GF) and negative cytoplasmic (Cy) and nuclear (N) ERA immunolocalization in the oocytes of the growing follicles (GF). Original magnification; A × 40, scale bar = 500 µm, B × 400, scale bar = 50 µm, C × 100, scale bar = 200 µm, D × 400, scale bar = 50 µm.

**Fig. 13:**
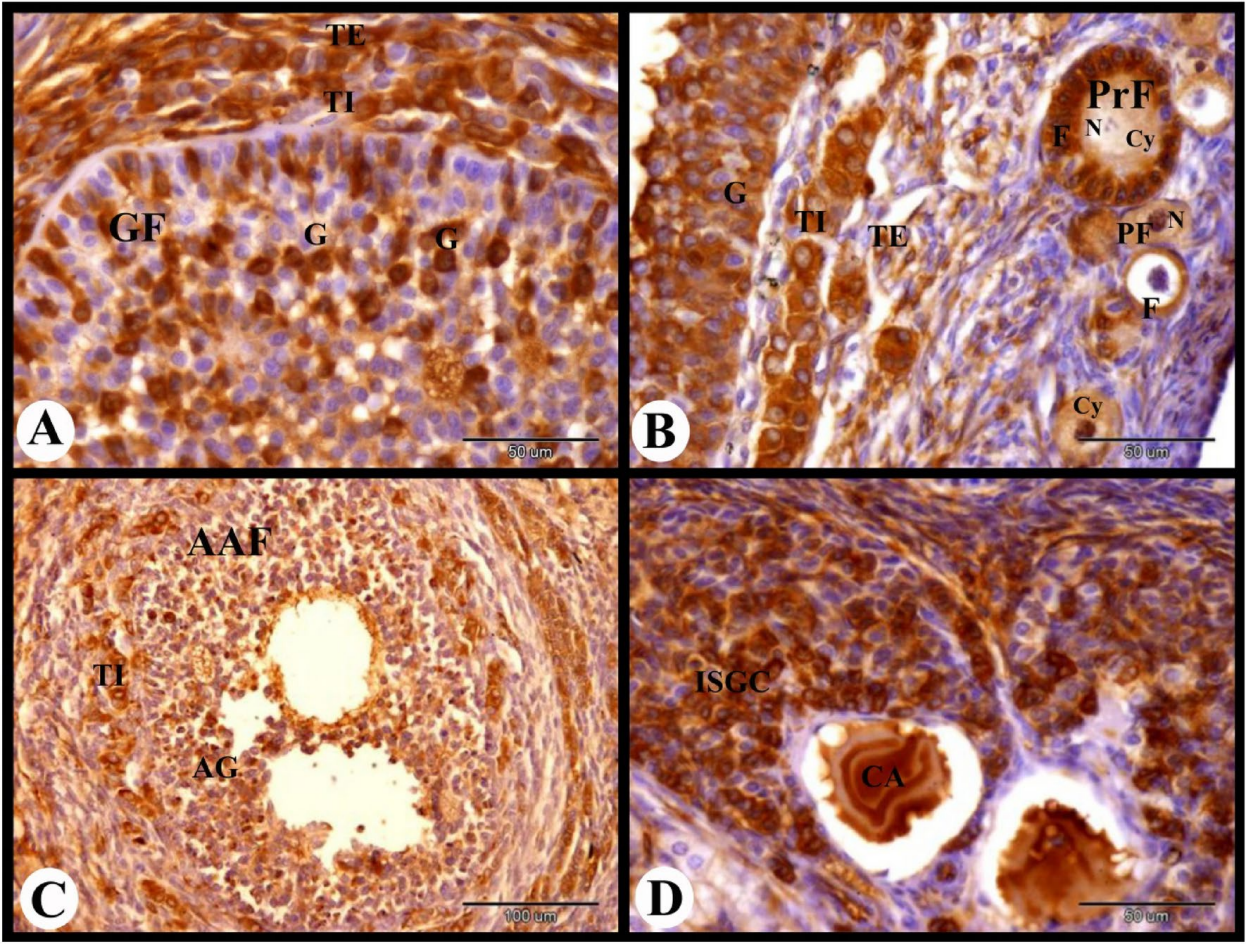
Photomicrograph showing ERA immunolocalization in the rabbit ovary 10 days of lactation. (**A**): Showing strong cytoplasmic and nuclear ERA immunolocalization in the granulosa cells (G), theca interna cells (TI) and theca externa cells (TE) of the growing follicle (GF). (**B**): Showing strong cytoplasmic and nuclear ERA immunolocalization in the granulosa cells (G), theca interna cells (TI) and theca externa cells (TE) of the large antral follicle, moderate cytoplasmic and nuclear ERA immunolocalization in the flattened follicular cells (F) of the primordial follicles (PF), moderate cytoplasmic (Cy) and nuclear (N) ERA immunolocalization in the oocyte of the primordial follicles (PF), strong cytoplasmic and nuclear ERA immunolocalization in the follicular cells (F) of the primary follicle, and moderate cytoplasmic (Cy) and nuclear (N) ERA immunolocalization in the oocyte of the primary follicles (PrF). (**C**): Showing weak cytoplasmic and nuclear ERA immunolocalization in the apoptotic granulosa cells (AG) and moderate cytoplasmic and nuclear ERA immunolocalization in the proliferated theca interna cells (TI) of the atretic antral follicle (AAF). (**D**): Showing strong cytoplasmic and nuclear ERA immunolocalization in the interstitial gland cells (ISGC) and corpora atretica (CA). Original magnification; A, B & D × 400, scale bar = 50 µm, C × 200, scale bar = 100 µm, D × 400, scale bar = 50 µm.

ERA immunolocalization in the rabbit ovary 10 days of lactation showed strong cytoplasmic and nuclear ERA immunolocalization in the granulosa cells, corona radiate cells, cumulus oopherous cells and theca folliculi cells of the preovulatory follicles. In addition negative ERA immunolocalization in the oocyte of the preovulatory follicles was observed. The small antral follicles showed moderate cytoplasmic and nuclear ERA immunolocalization in the granulosa cells (Fig. 12A). The fibroblast cells of the tunica albuginea showed weak cytoplasmic and negative nuclear ERA immunolocalization. While the interstitial gland cells showed moderate cytoplasmic and nuclear ERA immunolocalization. The primordial follicles showed weak cytoplasmic and negative nuclear ERA immunolocalization in the flattened follicular cells and negative cytoplasmic and nuclear ERA immunolocalization in the oocytes. The stroma cells showed moderate cytoplasmic and nuclear ERA immunolocalization (Fig. 12B). The ovarian surface epithelial cells showed strong cytoplasmic and nuclear ERA immunolocalization. The growing follicles showed strong cytoplasmic and nuclear ERA immunolocalization in the granulosa cells, theca interna cells and theca externa cells. While negative cytoplasmic and nuclear ERA immunolocalization in the oocytes of the growing follicles was demonstrated (Figs. 12C,D and 13A).

**Fig. 14:**
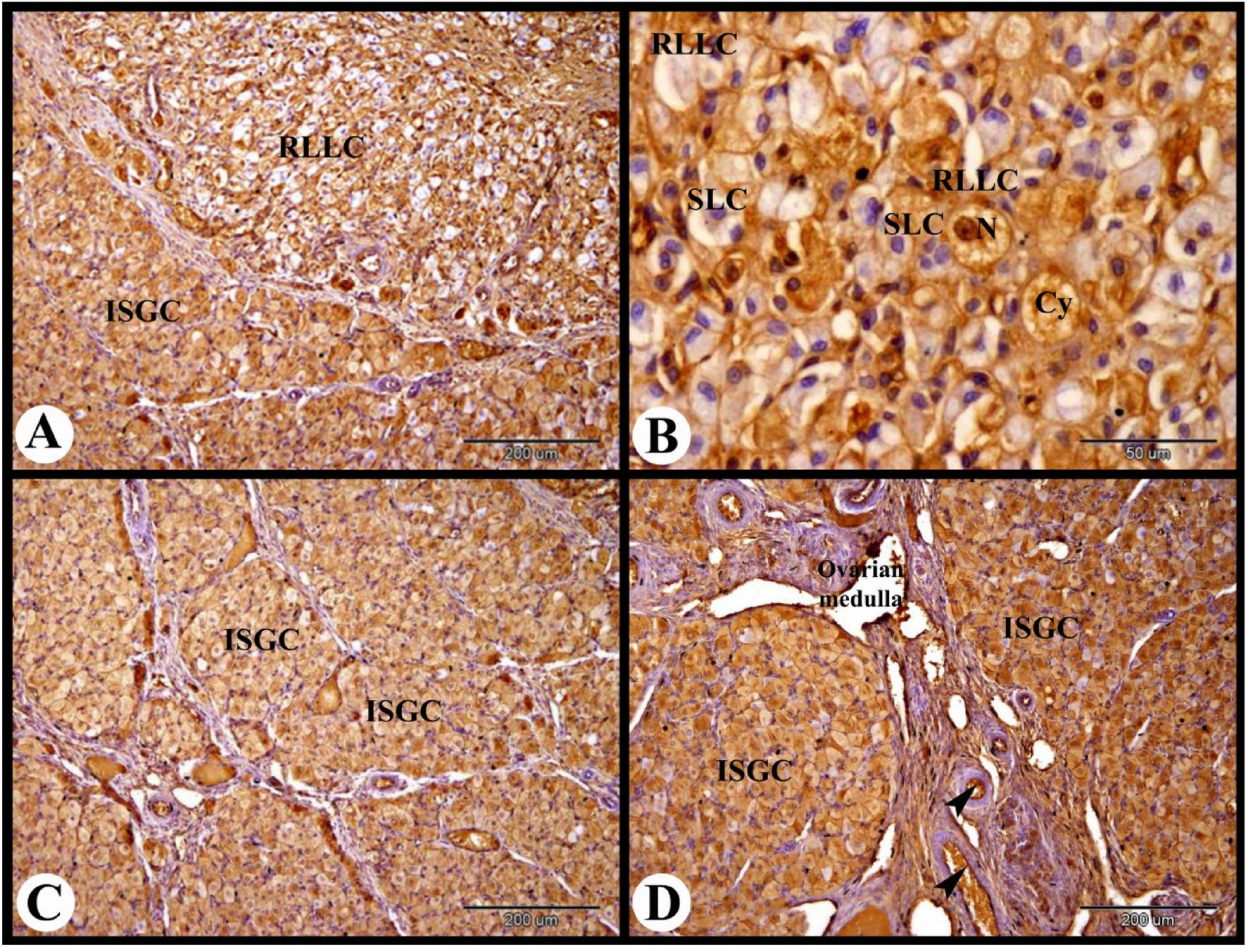
Photomicrograph showing ERA immunolocalization in the rabbit ovary 10 days of lactation. (**A**,**B**): Showing strong cytoplasmic and nuclear ERA immunolocalization in the interstitial gland cells (ISGC) and weak to moderate cytoplasmic (Cy) and nuclear (N) ERA immunolocalization in the regressed large lutein cells (RLLC), moderate cytoplasmic and negative nuclear ERA immunolocalization in the small lutein cells (SLC) of the regressed CL. (**C**,**D**): Showing strong cytoplasmic and nuclear ERA immunolocalization in the interstitial gland cells (ISGC) and endothelial cells (arrowheads) of the blood vessels of the ovarian medulla. Original magnification; A, C & D × 100, scale bar = 200 µm, B × 400, scale bar = 50 µm.

At this stage the primordial follicles showed moderate cytoplasmic and nuclear ERA immunolocalization in the flattened follicular cells and in the oocyte. The primary follicle showed strong cytoplasmic and nuclear ERA immunolocalization in the follicular cells, and moderate cytoplasmic and nuclear ERA immunolocalization in the oocyte. The large antral follicle showed strong cytoplasmic and nuclear ERA immunolocalization in the granulosa cells, theca interna cells and theca externa cells (Fig. 13B). The atretic antral follicle showed weak cytoplasmic and nuclear ERA immunolocalization in the apoptotic granulosa cells and moderate cytoplasmic and nuclear ERA immunolocalization in the proliferated theca interna cells (Fig. 13C). The corpora atretica showed strong cytoplasmic and nuclear ERA immunolocalization (Fig. 13D). The regressed CL showed weak to moderate cytoplasmic and nuclear ERA immunolocalization in the regressed large lutein cells and moderate cytoplasmic and negative nuclear ERA immunolocalization in the small lutein cells (Fig. 14A,B).

Interestingly, the rabbit ovary at 10 days of lactation showed abundant interstitial gland with strong cytoplasmic and nuclear ERA immunolocalization in the interstitial gland cells. As well as the ovarian medulla endothelial cells also showed strong cytoplasmic and nuclear ERA immunolocalization (Fig. 14C,D).

**Fig. 15:**
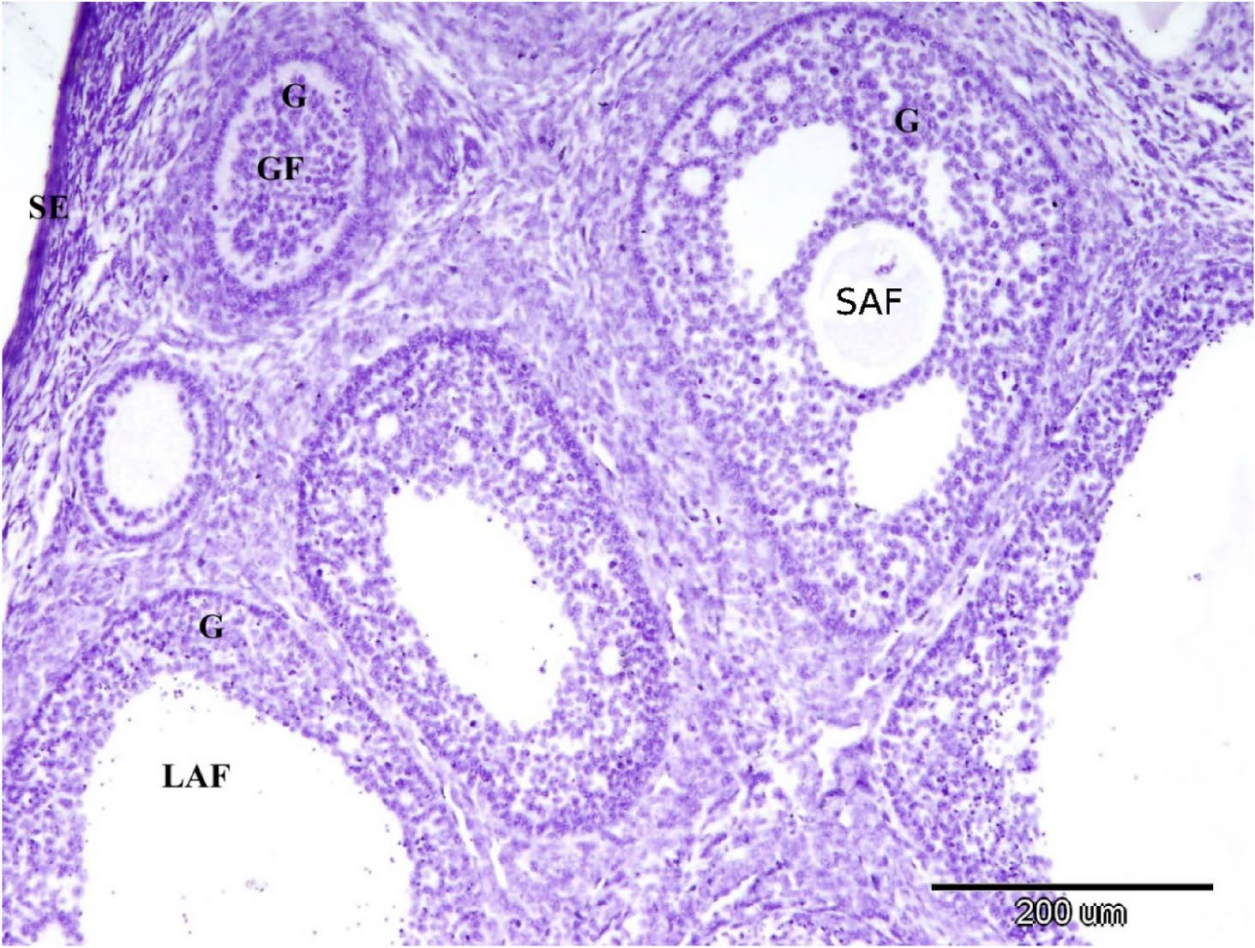
Control negative for ERA immunolocalization in the rabbit ovary; note the negative reaction in the ovarian surface epithelium (SE) and the granulosa cells of the growing (GF), small antral (SAF) and large antral (LAF) follicles. Original magnification × 100, scale bar = 200 µm.

Figure 15 showed the control negative for ERA immunolocalization in the rabbit ovary with negative reaction in the ovarian surface epithelium and the granulosa cells of the growing, small antral and large antral follicles.

The overall results of ERA immunorxpression in rabbit ovarian structures during pregnancy, after parturition and during lactation were summarized in Table 1.

**Table 1:** Showing ERA immunoexpression in rabbit ovary during pregnancy and lactation.

Stage	Main follicles		CL	Interstitial gland cells	
	Type	ERA	ERA	Amount	ERA
12 h	Preovulatory	+++	No CL	Small	+
3 days	Antral	+++	+	Small	+
7 days	Antral	+++	++	Small	+
14 days	Antral	+++	+++	Moderate	++
Parturient	Preovulatory	+++	+++	Moderate	++
10 days lactation	Preovulatory	+++	+	Large	+++

+++strong, ++moderate, +weak.

## Disscussion

In the current study, the cellular distribution of ERA in the rabbit ovary during pregnancy, postpartum and during lactation was investigated. Very limited studies investigated ERA in rabbit ovary during pregnancy and lactation. The differential expression of ERA and ERB is great in the ovarian tissues and cells^14^. The ovary of rat contains both ERs (ERA and ERB) subtypes, predominating ERB over ERA in granulosa cells^15^. In primate and human ovaries, ERA is diffusely expressed in thecal, interstitial, and granulosa cells of antral follicles, while ERB is predominantly located in granulosa cells^16^. In the rabbit ERA has a specific function during early pregnancy^17^.

By attaching itself to DNA sequences, E2 can penetrate the plasma membrane and interact with intracellular ER to directly affect cells. There are two types of E2-mediated signaling events: genomic and non-genomic. The migration of estrogen receptor complexes to the cell nucleus and direct contact with chromatin at particular DNA sequences known as estrogen response elements (EREs) are examples of genomic impacts. ERE sequence elements are absent from several other genes that are controlled by estrogen receptors. Conversely, non-genomic effects entail the indirect control of gene expression via a range of intracellular signaling pathways^18^.

E2 and its receptors play critical roles in folliculogenesis, the process by which ovarian follicles develop and mature. This process involves several stages, from primordial follicle recruitment to the formation of a mature follicle capable of ovulation. E2, acting through its receptors (ERA and ERB), influences key cellular and molecular events during this process, especially in the granulosa cells, theca cells, and oocytes^19^.

The immunolocalization of ERA in rabbit ovary during pregnancy and lactation demonstrated differential ERA immunolocalization in various ovarian structures. ERA is primarily expressed in the reproductive tissues (e.g., uterus and ovary), bone, white adipose tissue, kidney, liver, and breast^20^. A published review recommends that ERα mRNAs or proteins are expressed by mature immune cells and hematopoietic progenitors^21^. Weak to moderate ERA immunolocalization in the primordial and primary follicles suggests a role for E2 in the early stages of follicle development, though less prominent compared to later stages. E2 may influence the initial recruitment of follicles and support their early growth. It control the primordial follicle pool and the size of the oocyte in mice^22^. Strong ERA immunolocalization in the ZP of growing follicles points to a potential role for E2 in oocyte development and ZP formation. The ZP is a glycoprotein layer surrounding the oocyte, essential for fertilization. It was found that the hardness and thickness of the ZP can vary in relation to elevated E2 levels^23^. E2’s role in oocyte maturation is more indirect, largely mediated by its effects on granulosa and cumulus cells, which support the oocyte^24,25^. Both ER subtypes are expressed in homogeneous somatic cells and the oocytes through perinatal ovary development in the hamster. Moreover, ERs expression and differentiation of somatic cells to granulosa cells depend on perinatal FSH action^26^.

During pregnancy and lactation the strong cytoplasmic and nuclear ERA immunolocalization in granulosa cells of growing and mature follicles suggests that E2, through ERA, plays a direct role in regulating granulosa cell proliferation and differentiation. Granulosa cells support oocyte development, and their proliferation is critical for the formation of large antral follicles, a hallmark of follicular maturation^22,27^. In mature follicles, strong ERA immunolocalization in the theca interna indicates E2’s involvement in androgen production, which granulosa cells subsequently convert to E2. This androgen-E2 interplay is vital for folliculogenesis. Strong ERA immunolocalization in granulosa and theca cells, as well as the observation of mitotic figures with negative ERA immunolocalization, implies that E2 is necessary for granulosa cell proliferation but not directly linked to the process of mitosis itself. E2 is also known to have an anti-apoptotic effect on granulosa cells, which may help prevent follicular atresia during the critical phases of follicular growth^28^. In swine ERA was acting via an autocrine/paracrine role in the regulation of the ovarian function during pregnancy and for the process of successful reproduction^29^.

When follicles have all the elements of the "two cell, two gonadotrophins" model, they are first seen to be able to produce E2 in the late preantral stage. Small antral follicles have aromatase activity, but their inability to create an androgen substrate for aromatization to E2 limits their potential to manufacture E2 at this stage of development^5,6^. Therefore, growth that surpasses the small antral stage is marked by an increase in aromatase activity and androgen synthesis, which leads to the production of follicular E2. Since the preovulatory has the highest capacity for testosterone aromatization and the largest granulosa cell population, it has the highest intrafollicular amounts of E2^6,30^.

E2 is synthesized by the granulosa cells of ovarian follicles under the influence of follicle-stimulating hormone (FSH), which stimulates aromatase, the enzyme that converts androgens to E2s. E2 exerts a paracrine and autocrine function within the ovary to promote follicular growth and maturation. Specifically, E2 enhances the proliferation of granulosa cells which is essential for follicular expansion and maturation. It promotes the expression of FSH receptors in granulosa cells, thereby increasing their sensitivity to FSH, which is crucial for the progression of folliculogenesis. It also stimulates the expression of luteinizing hormone (LH) receptors in the pre-ovulatory follicles, preparing them for the LH surge that triggers ovulation^31^.

Overall, ERA shows a varied but distinct expression pattern in different ovarian cells, with strong localization in steroidogenic cells (granulosa and theca cells) and weaker or absent expression in oocytes. The expression also appears to correlate with follicular development stages, with more pronounced immunolocalization in cells involved in steroidogenesis.

The atretic antral follicle showed strong cytoplasmic and negative nuclear ERA immunolocalization in the apoptotic granulosa cells and strong cytoplasmic and nuclear ERA immunolocalization in the proliferated theca interna cells. This decline in E2 signaling may contribute to the process of follicular atresia (degeneration of follicles that do not reach full maturity). The collective evidence across a range of species appears to unequivocally show that E2’s atretogenic effects are at least partially mediated directly on the ovary, presumably through contact with the nuclear ER^32^. It has been discovered that the local E2 released by the growing follicles may play a physiological effect in regulating other follicular atresia^33^.

The CL during pregnancy till parturition showed moderate to strong ERA immunolocalization in the large lutein cells, small lutein cells, fibroblast cells and luteal endothelial cells. By day six of pregnancy, the rabbit CL is an E2-dependent tissue, and a sufficient supply of E2 is essential to prevent pregnancy failure^34^. The rabbit’s CL is necessary for maintaining pregnancy and seems to need luteotropins; E2 from ovarian follicles^35^. It was found that during pregnancy, the rat CL coexpresses both ER mRNA species (ER alpha and ER beta). On the other hand, ER alpha mRNA rose from the beginning of pregnancy, peaked in the middle, and then sharply decreased prior to delivery^36^.

It was discovered that the ER complex mechanism mediates the significant steroidogenic effect of E2 on ovarian progesterone (P4) synthesis in pseudopregnant rabbits^37^. A strong modulator of gonadotropin-stimulated steroidogenesis is E2. E2 is the only substance needed by the seventh day post-ovulation to stimulate the CL of superovulated pseudo-pregnant rabbits to synthesize P4. E2 luteotropic effect most likely doesn’t happen at P-450scc in the rabbit CL; instead, it most likely happens at control points that govern the availability of stored cholesterol and/or its transport to or within the mitochondria for pregnenolone synthesis^38^.

It is commonly known that the antral follicles are the primary source of endogenous E2 which considers the main luteoteophic hormone necessary for the function of the rabbit CL^39,40^. It was found that luteal tissue in pseudopregnant rabbit had aromatase activity and capable of synthesis luteal E2 that had an autocrine luteotrophic role^40^. The luteotrophic action of E2 is to regulate the production of P4^41^. Previous research suggested that the luteotrophic complex in rabbit include LH as well as E2 for normal luteal function. LH may contribute to the luteotrophic complex, at least in part, by inducing luteal cells to produce more E2 and ERA^42^.

Moreover, one study showed that significant changes occur in E2 metabolism in the rabbit CL within 8 days after ovulation, and that these alterations result from maternal factors expressed systemically rather than by the effects of the developing conceptus expressed locally^43^. One study showed that E2 hormone in combination with P4 play a critical role in the morphological and morphometric changes in the ovarian and uterine tissues of rabbits. This changes are mediated by binding with their receptors that is crucial for improving reproductive competence and fertility potential^44^. The luteal P4 synthesis in rabbits depends on the E2 level throughout the second half of pregnancy. This indicates that E2 is thought to be essential for preserving P4 levels throughout the gestational period^2^.

ERA and ERB mRNA expression were immunolocalized in human corpora lutea during the human luteal phase next luteal release with exogenous hCG^45^. Further studies showed that ERA was immunoexpressed in the rat^36,46,47^ and rhesus monkey^48^ CL. In rat CL, ERA mRNA expression was increased from early pregnancy, reached a peak at midpregnancy, and remarkably declined before parturition^36^.

The bovine CL showed the highest mRNA expression for ERA in the early luteal phase, with a notable decline in expression in the mid, late, and regression phases. While during pregnancy low levels of ERA was measured^49^. ERA was suggested to be involved in the processes of formation, maintenance, and regression of feline^50^ and swine^29^ CL.

In postpartum rabbits, ERA expression is likely influenced by hormonal changes associated with the reproductive cycle and lactation. The expression of ERA in ovarian tissues is regulated by circulating E2 levels. Postpartum, fluctuating E2 levels due to lactational anestrus or resumption of estrous cycles may affect ERA expression^2,51,52^. ERA modulates the production of key steroid hormones such as E2 and P4, crucial for resumption of the estrous cycle postpartum. It may interact with other receptors like FSH and LH receptors to ensure proper ovarian functionality^53^. ERA signaling may protect developing follicles from atresia by promoting cell survival pathways, which is critical for restoring fertility postpartum^54^. As E2 levels increase postpartum, ERA mediates the hormonal signals required for ovulation and preparation for subsequent pregnancies. Postpartum, ERA may support the recruitment and maturation of secondary follicles as ovarian activity resumes^30^.

The immunolocalization of ERA in the rabbit ovary at 10 days of lactation reveals a detailed pattern of expression across different follicular stages and ovarian structures. ERA expression is observed in various ovarian cells, with strong cytoplasmic and nuclear localization in granulosa cells, theca folliculi, cumulus oophorus, and corona radiata cells of preovulatory follicles, while no ERA is detected in the oocytes of these follicles. Small antral follicles exhibit moderate ERA localization in granulosa cells. It was found that, on day 11 following parturition, more rabbits accepted the male than on day 21^34^. This may be due to presence of many preovulatory follicles and increasing ERA immunoexpression. Interestingly, the rabbit ovary during lactation showed abundant interstitial gland with strong ERA immunolocalization in the interstitial gland cells. These cells were thought to be originate from theca interna of atretic antral follicles and regressed CL theca lutein cells (small lutein cells) and help in maintain the ovarian functions^39,55^. The primary output of interstitial gland cells appears to be the production of androgenic steroids; in certain mammalian species, progestin and E2 are also produced^56^. The rabbit ovary’s interstitial glands secretes progestin which help to maintains pregnancy^39,57^.

The regressed CL in the rabbit ovary 10 days of lactation showed weak ERA immunolocalization in the regressed large lutein cells and moderate cytoplasmic ERA immunolocalization in the small lutein cells. This suggests that E2 signaling is not a major regulator of the CL regression, as the focus shifts to decreasing P4 production. When the CL reaches the end of its life, it goes through a regression process that causes it to leave the ovary and makes room for a new cycle^58^. In rabbit Cl decrease in E2 production and ERs expression mediate apoptosis through a shift in expression of bcl-2 family members^59^.

Interestingly, ovarian telocytes showed strong cytoplasmic and nuclear ERA immunolocalization. Telocytes were observed in ovary of mice^60^, rat^61^, rabbit^12^, and human^62^. The main functions of the ovarian telocytes may include: the nursing of stem cells, intercellular signaling, the regulation of hormone-dependent processes, immune surveillance, and microenvironmental maintenance^63^. Furthermore, stromal cells and ovarian endothelial cells are strongly positive for ERA expression. In ovarian stromal cells, 17β-estradiol promoted steroidogenesis^64^.

This immunohistochemical pattern of ERA distribution provides insights into ERA signaling during the post-lactational phase, revealing its involvement in the regulation of ovarian follicular development, luteal regression, and stromal cell activity in the rabbit ovary.

Our results were expressed in a strong, moderate, weak and negative immunostaining scale, however in future studies, the use of modern quantitative image analysis as simple computerized programs (such as Image J) or AI based modern quantitative image programs will conduct accurate measurements, quantify stain intensity, and provide precise measurements such as cell counting in digital slides. This will give accurate and clear illustration of the quantitative and descriptive ERA distribution during pregnancy and lactation.

## Conclusion

This study concluded that ERA had an essential role in the ovulation, folliculogenesis, lutenization and luteal regression in the rabbit during pregnancy and lactation. These finding are increasingly important for enhancing this animal’s reproductive success.

## Data Availability

The datasets used and/or analysed during the current study are available from the corresponding author on reasonable request.

## Abbreviations

ERs: Estrogen receptors
ERA or ERα: Estrogen receptors alpha
ERB or ERβ: Estrogen receptors beta
HCG or hCG: Human chorionic gonadotropin
ZP: Zona pellucida
LH: Luteinizing hormone
FSH: Follicle stimulating hormone
GnRH: Gonadotropin releasing hormone
P4: Progesterone
E2: Estrogen, estradiol 17beta, estradiol
CL: Corpus luteum
